# Sleep architecture and lying behavior in horses across age groups and two housing systems: an overnight non-invasive EEG study

**DOI:** 10.3389/fvets.2026.1898580

**Published:** 2026-08-06

**Authors:** Lisa Zimmer, Johannes Peter Schramel, Manolis Lyrakis, Dagmar S. Trachsel, Ulrike Auer, Michael Leschnik, Sabine Macho-Maschler, Monika Hotz, Hanna Wahl, Susanne Brunner, Rupert Palme, Jessika-M. V. Cavalleri

**Affiliations:** 1 Clinical Department for Small Animals and Horses, Centre for Equine Health and Research, University of Veterinary Medicine Vienna, Vienna, Austria; 2 Clinical Department for Small Animals and Horses, Centre for Small Animal Health and Research, University of Veterinary Medicine Vienna, Vienna, Austria; 3 Department of Biological Sciences and Pathobiology, Platform for Bioinformatics and Biostatistics, University of Veterinary Medicine Vienna, Vienna, Austria; 4 Department of Biological Sciences and Pathobiology, Centre for Biological Sciences, University of Veterinary Medicine Vienna, Vienna, Austria; 5 Zaza Textillösungen GmbH, Koblach, Austria; 6 Equine Internal Medicine, University of Veterinary Medicine Vienna, Vienna, Austria

**Keywords:** equine age impact, equine EEG, equine sleep, equine welfare, first-night-effect

## Abstract

Sleep is a crucial factor for the well-being of horses, yet assessing equine sleep in common housing conditions remains challenging. This study investigated the effects of changes in horses’ immediate environment and the influence of age on their sleep and resting behavior using a novel, non-invasive electroencephalography (EEG) mask. Ten horses from the teaching herd at the University of Veterinary Medicine Vienna were observed over four consecutive nights in two different housing systems (a large communal stable vs. two separate, adjacent individual stalls). Data collection included EEG recordings, video monitoring, electrocardiography for heart rate variability (HRV) analysis, and fecal cortisol metabolite (FCMs) measurements. The results showed significant differences in sleep stages and lying behavior between the two housing systems. Over time, the horses, in the single stable setting, exhibited an increase in REM sleep and lying-down frequency, while in the large barn setting, they showed longer duration of lying episodes, suggesting an adaptation effect. Younger horses exhibited more REM, N1 and N2 sleep, more complete sleep cycles and longer lying episodes compared to older horses. Objective stress monitoring via HRV analysis showed trends toward increased parasympathetic activity, while FCMs showed no significant changes. These findings suggest that housing conditions and age should be considered when assessing equine sleep as well as well-being. and reinforce the need for management practices that promote adequate rest. Further research is required to establish normative values and validate EEG protocols in equine medicine and to investigate the associations between sleep, stress, and well-being.

## 1 Introduction

Sleep is a critical yet often underestimated indicator of equine welfare (1–4), with important implications for health, performance, and quality of life. Across species, adequate sleep supports immune function, metabolic regulation, cognition, and overall well-being (5). In humans, insufficient sleep is associated with cardiovascular disease, metabolic disorders, and cognitive impairments (6). In horses, sleep deprivation has likewise been linked to poorer cognitive performance and altered behavior (7–9). Stress can markedly disrupt equine health, lying behavior, and consequently sleep patterns, underscoring the importance of appropriate management. Experimental evidence from other species highlights the significance of sleep loss, with prolonged deprivation leading to metabolic dysfunction, immune suppression, and even death in rats (10, 11), underscoring the welfare relevance of sleep.

Sleep in mammals is organized in cyclic patterns of non–rapid eye movement (NREM) and rapid eye movement (REM) sleep and is depicted in a species-specific hypnogram governed by circadian and homeostatic processes (1, 12–14). Alterations in sleep structure such as reduced duration, shifts in NREM/REM balance or increased fragmentation, may indicate stress and compromised welfare (2, 4, 15, 16). In humans, sleep architecture is assessed with polysomnography, which integrates electroencephalography (EEG), electrooculography (EOG), and electromyography (EMG). According to the American Academy of Sleep Medicine (AASM) criteria, sleep is divided into three NREM stages (N1–N3) and REM sleep (17). Sleep architecture changes across the lifespan, with age associated changes including reduced slow-wave sleep (SWS; N3) and reduced REM sleep, greater sleep fragmentation and altered neuroendocrine regulation (18–22). Sleep composition also varies throughout the night, with NREM predominating in the first half of the night and REM sleep in the second half of the night (19, 23).

In horses, one-to-one transfer of AASM N1–N3 terminology is not fully validated. So far, four vigilance states have been described using EEG in horses: wakefulness, drowsiness, slow-wave sleep (SWS), and paradoxical sleep (PS; REM) (16, 24–31). In the equine EEG, the wakeful state is mostly characterized by high-frequency beta waves (13–30 Hz) and low amplitudes, whereas the transition to sleep (N1/Light Sleep) involves a frequency reduction into the theta range (3–7 Hz) (4, 24). Slow Wave Sleep (SWS) or N3 is defined by dominant delta waves (< 4 Hz) with high amplitudes. REM sleep presents a desynchronized EEG activity like wakefulness or drowsiness (low voltage, mixed frequency), accompanied by rapid eye movements and minimal muscle tone (4, 26). In the present study, we adopt a pragmatic mapping while explicitly defining stage criteria: we refer to N1 as drowsiness (light sleep; predominantly theta), N3 as SWS (high-amplitude delta), and REM as paradoxical sleep (low-voltage mixed-frequency EEG with REMs and muscle atonia). We label N2 as intermediate NREM stage, with decreased frequency (mixed theta–delta activity) and increasing amplitudes compared to wakefulness and the N1 stage. Sporadic sleep-spindle-like graphic elements can also be seen. All staging decisions are grounded in the forecited literature and tailored to species-specific sleep organization.

Like other large prey animals, horses exhibit polyphasic sleep with multiple short episodes distributed over 24 h and a total daily sleep duration of approximately 3–5 h, predominantly during the night (4, 26). Horses spend 15–25% of their day resting (26). While SWS may occur in standing posture, REM sleep requires recumbency due to muscle atonia (32). Consequently, factors that reduce lying behavior directly constrain REM sleep expression. Recumbency is influenced by environmental security, social context, bedding quality, familiarity with surroundings, and physical comfort (27–36). Horses in unfamiliar environments may delay lying for several days, suggesting that perceived risk modulates sleep (36). Pain is another critical determinant: musculoskeletal pain can suppress recumbency and REM sleep an lead to sleep deprivation, paralleling observations in humans (2, 3, 5, 27, 37–39). Developmental and aging effects are incompletely characterized in horses, but foals sleep more than adults, with a higher proportion of SWS and less REM (27). Comprehensive lifelong data and age specific sleep profiles remain limited.

Despite its growing importance for animal welfare research, equine sleep research still relies heavily on observational methods that do not allow for a precise distinction among sleep stages. Non-invasive telemetric EEG or full polysomnography in freely moving horses has been limited to specialized settings, restricting ecological validity (15). Improved, field-deployable methods are needed to quantify stage-resolved sleep in conventional housing and to assess how environmental management and age shape sleep architecture.

As pain and stress are established contributors to sleep disturbance across species (38–43), incorporating objective stress measures for sleep evaluation is essential. In horses, standardized ethological assessments such as the Horse Grimace Scale, facial expressions, posture, and broader ethograms are commonly used (44–51) and can be complemented by physiological metrics. Heart rate variability (HRV) provides a non-invasive index of autonomic regulation; time- and frequency-domain parameters inform on sympathetic–parasympathetic balance and respond to environmental conditions and acute stressors such as transport (52–59). Root Mean Square of Successive Differences (RMSSD) reflects parasympathetic activity and Standard Deviation of the NN Intervals (SDNN) represents overall variability; reductions in these indices are associated with stress responses (60). Short-term parasympathetic activity differences are reflected in the changes in RMSSD and SD1 (the width of the ellipse, represents short-term variability), while SD2 (the length of the ellipse, represents long-term overall variability) reflects the balance between sympathetic and parasympathetic systems (60, 61). In sleep research, HRV enables examination of nocturnal autonomic modulation in relation to behavioral and electrophysiological states.

The hypothalamic–pituitary–adrenocortical (HPA) axis can be non-invasively assessed via fecal cortisol metabolites (FCMs) using enzyme immunoassay, and reflect circulating cortisol with a species-specific delay (62). In horses, FCMs typically peak about 24 h after ACTH administration, with basal concentrations around 0.7–10.7 ng/g (53). FCMs monitoring is used to evaluate the effect of environmental, health and housing conditions. Horses kept in single stalls without direct contact show higher FCMs concentrations than those in semi-contact, paired or group housing (63–65). While HRV and FCMs provide objective insights, they differ in temporal resolution, and FCMs may not capture rapid, acute changes during sleep.

To address methodological constraints, we developed and validated a non-invasive telemetric EEG mask enabling overnight recordings in freely moving horses under normal housing conditions. Using this approach, we addressed three primary objectives: (i) determine the influence of housing environment (shared barn vs. individual stall) on sleep architecture and lying behavior; (ii) assess adaptation across four consecutive nights to evaluate potential first-night effects; and (iii) compare sleep parameters between younger and older adult horses. Secondary objectives were to evaluate the feasibility and tolerability of the EEG mask and to explore associations between sleep architecture and physiological stress indicators (HRV and FCMs).

We hypothesized that: (1) sleep, particularly REM and recumbency would be reduced upon first exposure to a novel housing condition and would increase with acclimation; (2) shared housing would facilitate recumbency and increase REM expression compared with individual stalls; (3) younger horses would show greater total sleep time and a higher proportion of REM than older horses; and (4) nocturnal HRV would increase as sleeping behavior intensified and across consecutive nights, reflecting enhanced parasympathetic dominance, whereas FCMs would reflect housing-related stress with a delayed time course. By integrating behavioral (discomfort score, lying behavior observations), physiological (HRV analysis, FCM measurement) and electrophysiological (EEG, ECG) measures, our data advances the understanding of the effect of housing management and age on sleep and potential welfare in a group of horses.

## 2 Materials and methods

### 2.1 Horses, settings and equipment

The study initially included 12 horses, from the Vetmeduni-owned teaching herd (Supplementary Table S1); however, one horse had to be excluded due to uncooperative behavior and another due to hospitalization during the study period. The study was approved by the Ethics and Animal Welfare Committee of the University of Veterinary Medicine, Vienna in accordance with the University’s guidelines for Good Scientific Practice. Horses were paired based on individual preferences and herd hierarchy to minimize stress, and a random draw (pairs labelled A–F) determined the order in which each pair underwent the experimental protocol.

**Figure 1:**
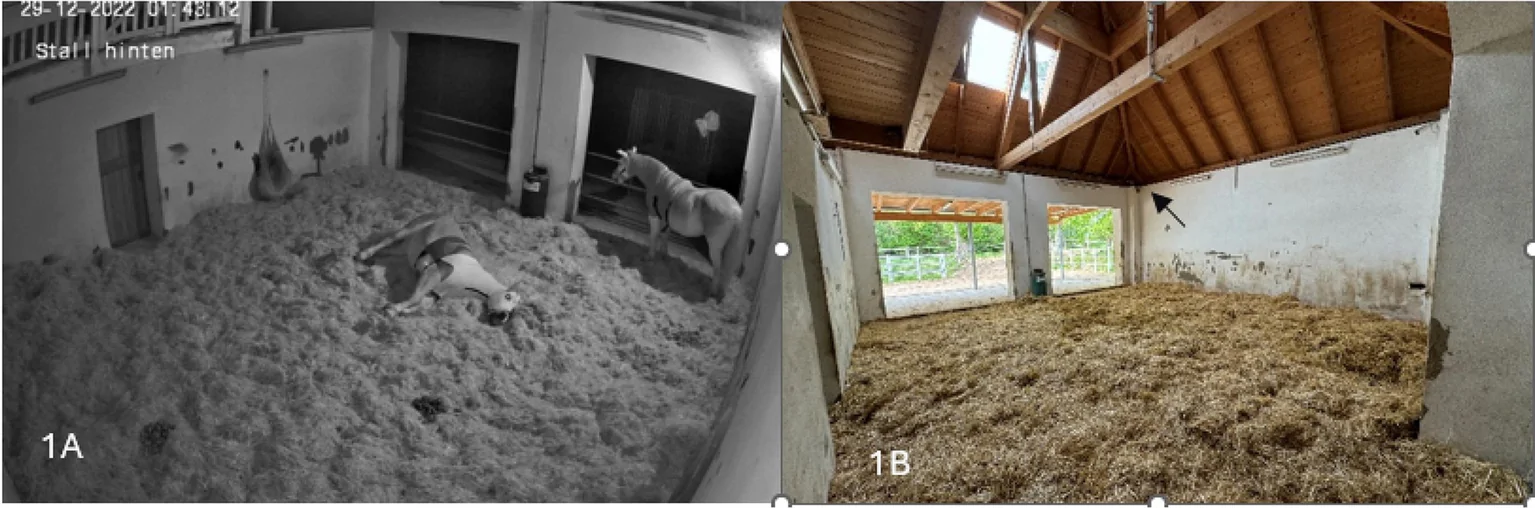
**(A)** Lying area Setting 1 from the perspective of the camera located in the back of the barn. **(B)** Photograph taken from the Lying area Setting 1, the black arrow marks the location of the camera in the front of the barn.

**Figure 2:**
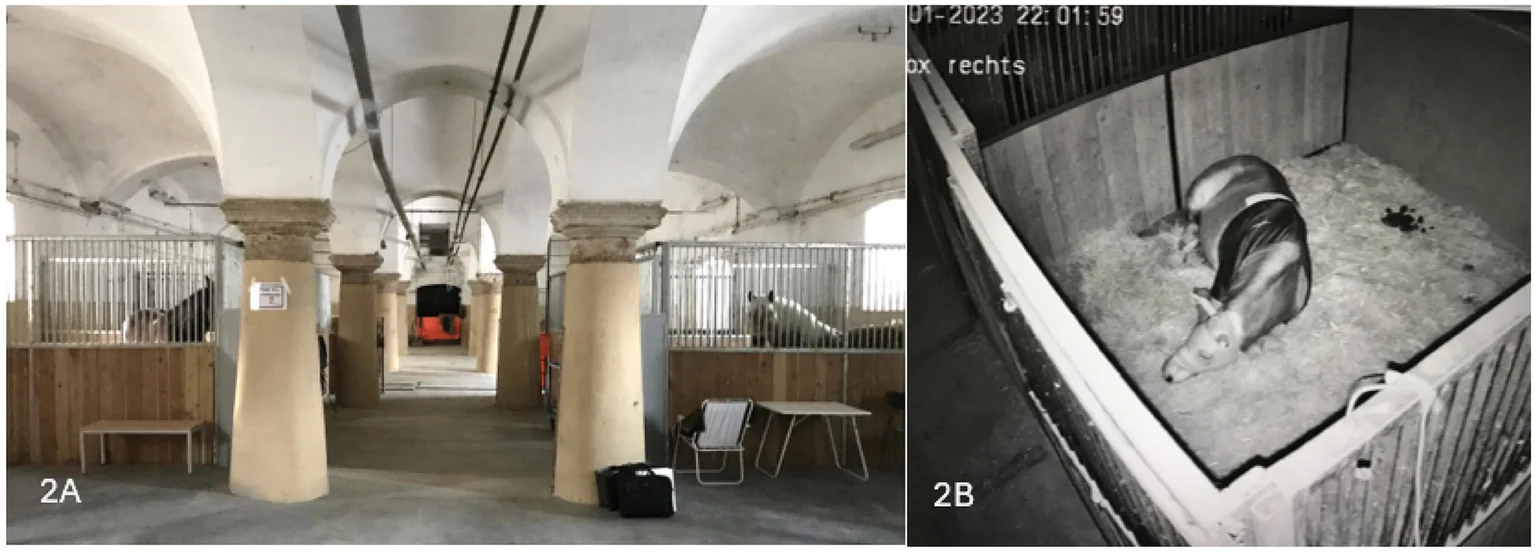
(**A**) Photograph from the entrance to the stable used as Setting 2. **(B)** Picture from the camera located at the right single unit stable.

Two housing settings were used to evaluate sleep and lying behavior. Setting 1, the shared barn, is part of the teaching herd’s standard housing facilities and includes two large lying areas accessible from an external pen. One lying area (69.41 m^2^, 8.5 m × 8.1 m) was used for the experiment and was therefore (Figures 1A,B; Supplementary Figure S1) closed off with gates, allowing visual and social interaction with other herd members. The bedding type used in this area was deep litter bedding with straw, in quantities sufficient to account for consumption associated with foraging behavior. A drinking trough and two hay nets were available. Setting 2, the stable units, are located within a multipurpose facility, near Setting 1 and consist of five 3,5 m × 3,5 m stalls, bedded with a regular layer of straw, which was completely removed and exchanged daily. The horses of each pair were placed in stable units opposite each other. Typically, these stable units were used for lectures, training, or isolating ill horses (Figures 2A,B; Supplementary Figure S2). All horses were familiar with both settings prior to the study. For the analysis the horses were further stratified into a young group (≤10 years) and an older group (≥18 years), with six horses in the old group category and 4 in the young group category.

Horses underwent a habituation period, 1 month before the start of data collection, to become accustomed to experimental procedures and equipment.

### 2.2 EEG and video setup

**Figure 3:**
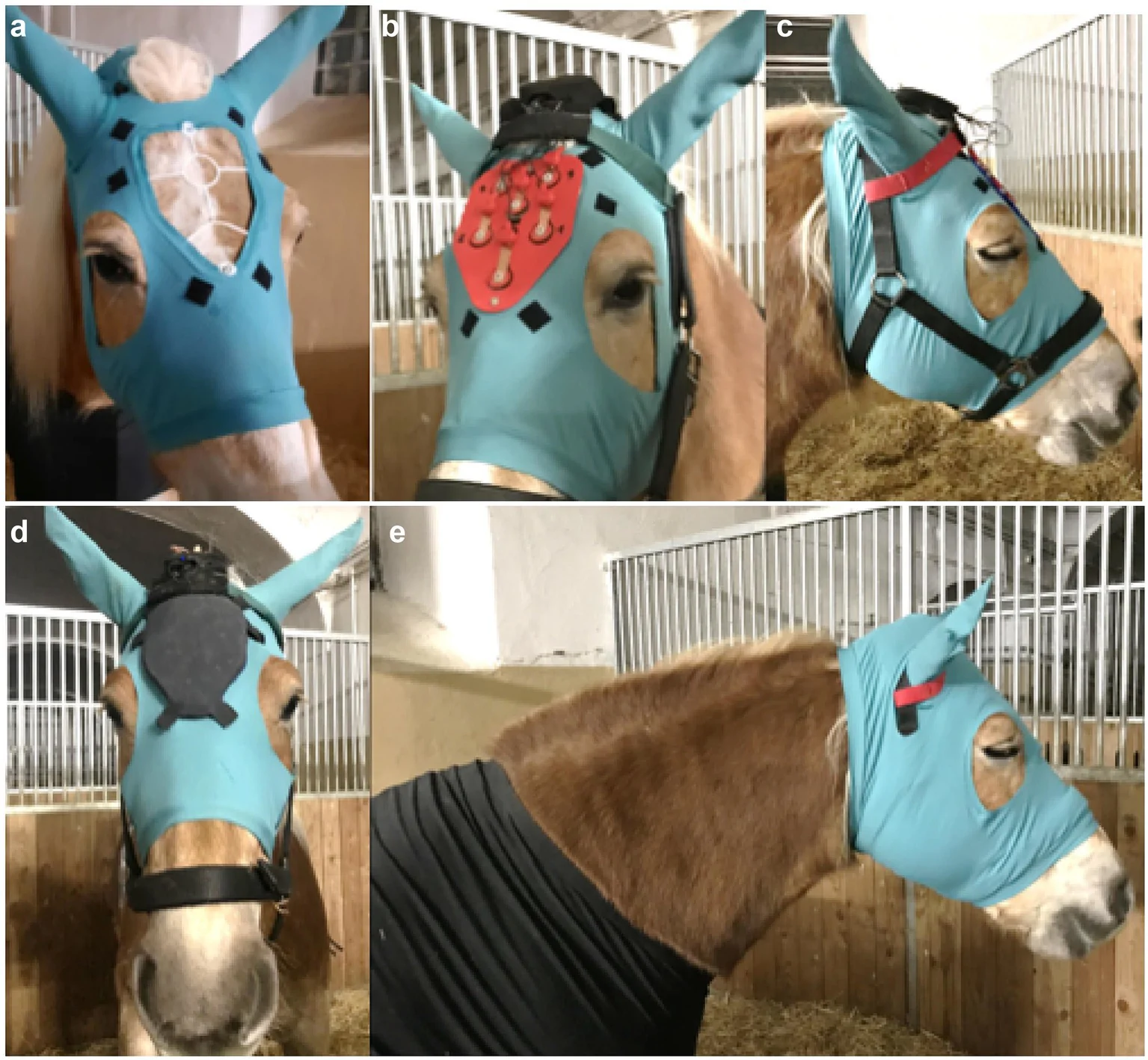
EEG mask setup: **(a)** mask with sewn in frame, **(b)** flexible plate with electrodes clipped onto frame, **(c)** cable connection between plate and Ganglion, **(d)** soft padding to maintain electrode-skin contact, **(e)** protective over-mask.

A custom-designed, non-invasive EEG face mask for horses (Zaza Textillösungen, Koblach, Austria) was used to record brain activity overnight. The mask was made from an elastic garment and incorporated a 3D-printed frame at the forehead to fix a flexible electrode plate with six electrodes (channels 1–4, reference and ground, as seen in Figure 3). The rubber electrodes (Soft Pulse, Datwyler Swiss) were connected to an OpenBCI Ganglion board (OpenBCI Inc., Brooklyn, NY, United States) housed in a custom designed casing with a 500mAh LiPo battery and a charger board. The box was placed in a pocket between the ears fixated on the halter (all together weighing 710 g). The electrodes for the four channels were placed in a 60×60 mm square, with the ground electrode in the center. The reference electrode was placed 80 mm distal from it.

Prior to mask attachment, the forehead was cleaned with a brush and clipped for electrode placement. The electrode attachment sites were then cleaned with gauze compresses (ES-Kompressen, Paul Hartmann AF, Germany) and soap (Softaskin Pure B Braun, Germany), rinsed with gauze compresses and water and dried. To lower the contact resistance, 5% salicylic acid in ethanol was used. A paste enhancing electrical conduction (Ten20 Conductive, Neurodiagnostic Electrode Paste, Weaver and Company, United States) was subsequently applied on the skin. Once the mask was in place, the electrode plate was attached and covered with a foam pad to apply continuous pressure and secure a durable electrode contact.

Electrode impedance was checked prior to and at the end of each recording. The impedance measurements in the OpenBCI GuI software were reported in kΩ and displayed in a color-coded format for ease of assessment. An impedance of less than 10 kΩ was considered very good and marked in dark green; impedances between 10 and 100 kΩ were marked light green and impedances above 100 kΩ were red. An impedance below 10 kΩ was targeted and achieved in most of the recordings (Supplementary Table S2). Impedance checks were repeated throughout the night. The Ganglion board transmitted the EEG signals with a sample rate of 200 Hz via Bluetooth to a laptop executing the OpenBCI- GUI software for real-time visualization, impedance evaluation and recording. Timestamps were synchronized manually with video timecode by the first author (LZ).

On recording nights, the mask and electrodes were fitted approximately 30–60 min before data acquisition commenced to allow the horses to acclimate to the equipment and minimize any transient effects associated with handling and equipment placement.

Video monitoring was conducted using high-resolution outdoor cameras with night vision (IPCB64521 IP Tube 4 Mpx, ABUS). Cameras were positioned to capture the entire lying area or individual single-unit stalls as appropriate (Figures 1A, 2B).

For artifact handling and quality control, movement artifacts, signal saturations, and Bluetooth dropouts were identified during *post hoc* review. Disconnections or severe artifacts which could not be identified as movement associated via video analysis resulted in the affected epochs being excluded from analysis. For each night, the amount of usable EEG data per horse and setting was recorded to document data yield and signal quality.

### 2.3 ECG setup

**Figure 4:**
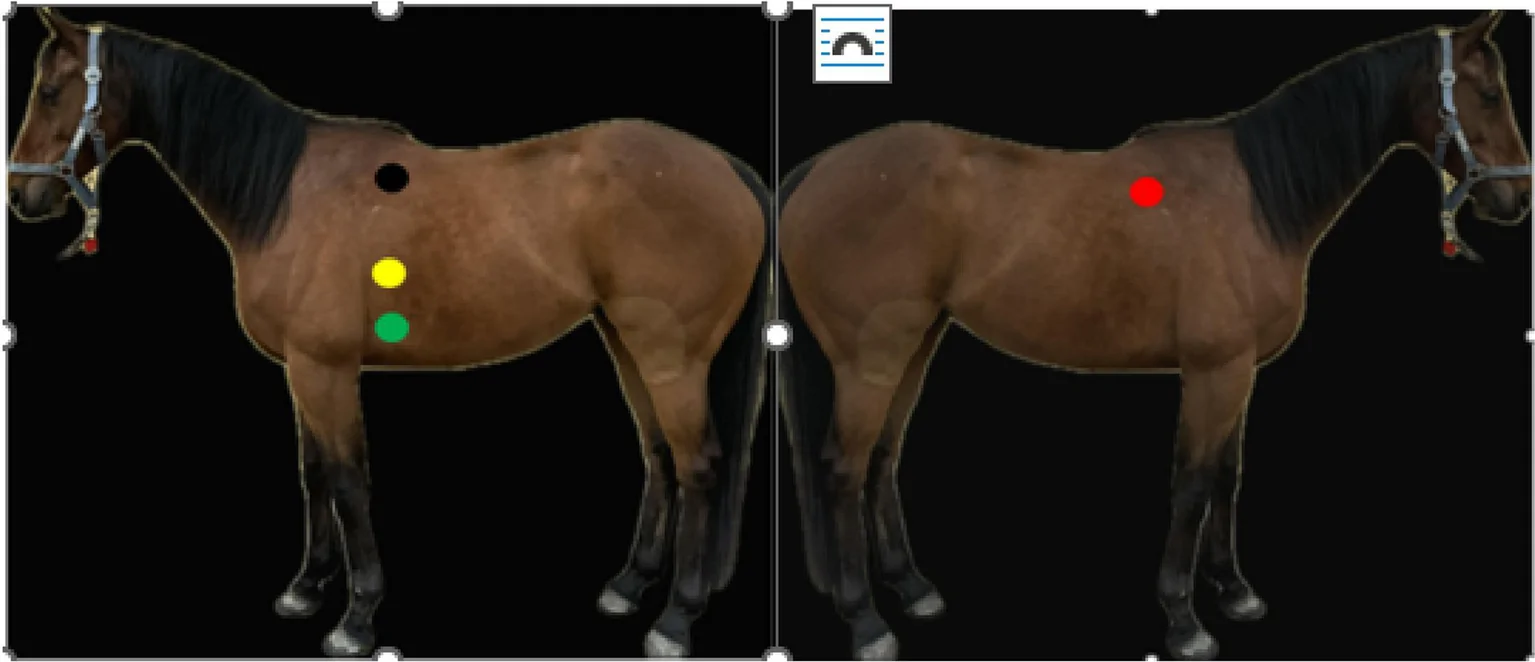
ECG electrode placement: Green: Left thorax, girth area at elbow height, Yellow: Dorsal to green, Black: Left saddle area, caudal to scapula, Red: Right thoracic saddle area.

Electrocardiography (ECG) was recorded using the Televet System (Engel Engineering Services GmbH, Germany) for subsequent heart rate variability (HRV) analysis. Recording followed a modified base-apex configuration using four surface electrodes (Figure 4). The skin at each electrode site was clipped and thoroughly cleaned with soap (Softaskin Pure B Braun, Germany) and ultrasound gel (Ultraschallgel, Gello GmbH Geltechnik, Germany) was applied before electrode placement. Electrodes were secured with adhesive foam bandage (Snögg, Animal Care, Norway) and a padded surcingle. The Televet 100 recorder was attached to the surcingle, and a sleezy on top provided additional protection. Electrode wires were routed along the girth and under the surcingle to minimize movement artifacts and snagging.

The ECG sampling rate was 500 Hz and a notch filter at 50 Hz was used. Data quality was regularly controlled by visual inspection. Timestamps were synchronized manually with video timecode by author (LZ). A maximum R-R interval deviation of 20% was accepted. Signal performance was considered adequate when P, QRS, and T waves were distinguishable.

Electrodes and cables were inspected after each night for displacement or skin irritation. Any redness or abrasion was documented and received appropriate veterinary care. No additional restraint was used beyond the surcingle and sleezy. Each morning the adhesive foam bandages covering the electrodes were carefully removed using skincare oil (Baby Pflege-Öl, Nivea, Germany).

### 2.4 Experimental protocol

Data collection took place from 26 December 2022 to 3 March 2023, to minimize seasonal confounding effects. Additionally, during data obtainment, the temperature was recorded daily (as seen in Supplementary Table S3). Each pair was evaluated during eight nights, with four consecutive nights in each housing setting. We used a randomized, counterbalanced crossover: each pair experienced both settings, with sequence (Setting 1 → 2 vs. Setting 2 → 1) randomized across pairs. Each recording night ran from 6 p.m. to 6 a.m. (12 h). Horses rejoined the herd at 7 a.m. the following morning (Supplementary Table S4).

Until 3 p.m. horses were roaming freely in the herd’s regular enclosure. At 3 p.m. they were brought to their respective setting. From 4 p.m. preparations took place (skin preparation; application of EEG mask and ECG; equipment function checks), aiming for a consistent start of recordings at 6 p.m. Weekends (Friday morning to Monday early afternoon, about 77 h) served as a “wash-out” period, during which horses returned to their usual management and no recordings were performed.

All data collections, preparations and routine checks were performed by the first author (LZ) to standardize handling across nights and settings. Horses received hay in nets and on the floor, had ad libitum access to water and were bedded on straw.

Shared-barn setting: A gallery above the lying area housed the technical setup. Laptops were placed on a desk, and Bluetooth dongles for electrophysiological data reception were suspended from the gallery above the horses. A small desk light remained on overnight. The gallery was accessed via stairs from a hallway. First Author (LZ) remained in the gallery area during the periods 18:00–22:00, 23:30–02:45, and 05:45–06:00 to periodically monitor the computer screen and detect potential transmission failure. Horses were only approached in the barn, when signal interruption or electrode mal function was identified and electrode inspection or adjustment was required.

Single stall setting: Laptops were placed on a desk in front of the stalls, within the horses’ line of sight, and covered with a white sheet to protect from dust. Continuous monitoring software remained active, and the laptop screens provided the sole source of illumination in the otherwise dark barn. In-person checks occurred approximately every 2 h overnight.

Horses were monitored for signs of distress (nervous behavior), equipment intolerance (scratching, rubbing, biting) or skin irritation (swelling, scaling, sensitivity). Predefined criteria for pausing or terminating a recording were excessive artifacts or equipment displacement. To avoid disturbing sleep, horses were not approached when lying or sleeping even if ECG signals were artifact-contaminated; rather electrode inspection and corrections were performed once the horse awoke. All adverse events and protocol deviations were logged and considered during data-quality review.

### 2.5 Fecal sample collection

Fecal samples were collected to measure cortisol metabolites. Baseline samples were collected for each horse on three mornings (6–10 a.m.) and three evenings (4–8 p.m.) in the week prior to the each horse undergoing the experimental measurements. During the setting exposures, samples were obtained twice daily (morning and evening) from Monday to Friday. Each sample was identified by horse ID, date and time and setting, and placed into prelabelled containers. To minimize contamination, fresh boli were collected immediately after observation of voiding. Collectors wore disposable gloves and used clean gloves for each sample to avoid cross-contamination. Approximately 20 g of feces were transferred to each container. Directly after collection, samples were frozen at −20 °C. At completion of the trial, they were transferred via cooler box to the laboratory’s freezer and stored at −20 °C until analysis.

### 2.6 Discomfort scoring

A previously described discomfort scoring system (44) was used to assess potential pain or discomfort. The scale ranges from 0 to 19, with higher scores indicating greater musculoskeletal discomfort. Scoring was performed by the first author, who was trained in the ethogram and scoring rubric. Horses were observed in their assigned environment (Setting 1 or 2) at rest and during spontaneous movement, as specified in the scale. Each horse was scored three times, once at the start of the week (Monday) in each setting and during baseline weeks. Observations were made from a safe distance to minimize interference, and each assessment lasted approximately 5 min.

### 2.7 Data and laboratory analyses

#### 2.7.1 Sleep behavior analysis

EEG data were saved as CSV files using OpenBCI-GUI software (v 5.1.10 Firme, Land) and converted to EDF files using EDF Browser (Version 2.05 64-bit, Copyright (C) 2007–2023 Teunis van Beele, Land). The files were then imported into Domino Software (Version 3.0.0.6, SOMNOmedics AG, Germany). As the software’s automated classifiers were developed for human polysomnography and demonstrated insufficient sensitivity and specificity for equine signals, automated sleep stage classification was disabled, and all recordings were scored manually.

Thirty-second epochs were classified based on EEG frequency, amplitude, and recognizable patterns (e.g., spindles), and the corresponding time-locked video footage of the horse’s posture and behavior. Epochs with significant artifacts or transmission disturbances were marked as “not interpretable” and excluded from further analysis. If just one channel was impaired, the remaining clean channels were classified. Only epochs with identifiable features were included in stage counts and downstream analyses. Sleep stages were categorized as wakefulness, N1 (light sleep), N2 (intermediate NREM Stage), N3 (SWS), and REM sleep. An epoch was assigned a specific stage if at least 16 s of the 30-s window met the criteria for that stage.

An epoch was annotated as “wakefulness” if over 50% of the signal showed frequencies within the alpha and beta spectrum. Additionally, the information regarding posture and physical activity obtained from the video footage was used to confirm a matching activity level. This stage was often strongly artifact contaminated, as most horses spend a significant amount of time foraging. Regularly occurring blink artifacts or eye movement-associated artifacts were also present.

The N1 stage was characterized by a reduction in EEG frequency compared with wakefulness. Graphical elements described in the human literature, such as vertex waves, could also be present. Movement-related artifacts were absent, and eye movements were reduced.

The N2 stage was characterized by the presence of sleep spindles and predominantly theta-wave activity (4–8 Hz). Horses assumed variable body positions during N2 sleep, ranging from standing quietly with a relaxed head and neck posture to sternal or lateral recumbency.

N3 represented the deepest stage of NREM sleep with at least 20% of an epoch containing slow-wave activity characterized by low-frequency Delta oscillations (0.5–4 Hz) with high peak-to-peak amplitudes. Horses frequently transitioned into recumbency during this sleep phase, although N3 sleep could also occur while standing with a relaxed and lowered head and neck.

Rapid eye movement (REM) sleep was identified primarily by the characteristic eye movements detected by electrodes positioned adjacent to the eyes. In addition, REM sleep was associated with mixed-frequency activity and generally low EEG amplitudes. During REM sleep, horses rested either in lateral recumbency or in sternal recumbency with the head resting on the ground and the neck on or near the ground.

Intra-observer repeatability analysis was performed using the Cohen’s kappa provided by Domino Software to ensure consistency in sleep stage recognition. Two randomly assigned nights from two different horses were re-scored.

EEG time stamps were aligned to the video timecode at the start of each session via manual correction, ensuring posture verification for each epoch. Manual scoring was performed by the first author, who was familiar with equine EEG features and the study ethogram.

The proportion of included epochs per horse/night/setting was tracked and reported (Supplementary Table S5).

#### 2.7.2 Lying behavior analysis

Video recordings (6 p.m. – 6 a.m.) were used to score lying behavior, including the duration and frequency of recumbent episodes. Recordings were analyzed by direct observation to distinguish active behavior, drowsiness, and lying behavior during sleep phases. Video data were cross-referenced with EEG readings to identify sleep stages. Lying duration was defined as the time from lying down to standing up. All positional changes during recumbency were documented, including sternal recumbency and lateral recumbency, with or without the head and neck resting on the ground. Rolling immediately followed by rising was not classified as recumbency. Artifacts seen on EEG were aligned with video data and checked for signs of active behaviors such as foraging, chewing or rolling. A lowered head and neck position while standing still, with or without a leg resting position was observed and was considered a behavioral indicator of drowsiness (66).

These behavioral data were cross-referenced with EEG recordings to identify sleep stages. Derived variables aligned with EEG analyses included:

(I) number of lying cycles per night,
(II) duration of lying per cycle,
(III) total lying duration per night,
(IV) time-of-night distribution (before midnight vs. after midnight), and.
(V) time spent in sternal and lateral recumbency.

#### 2.7.3 ECG

Real-time ECG data were transmitted via the Televet 100 system, Televet II Recorder (Engel Engineering Services GmbH, Germany) and reviewed using Televet software (ECG Software Version 7.0.2 Build:004, Copyright ^©^ 2006–2019 Engel Engineering Services GmbH, Germany). Five-minute artifact-free ECG segments (300–330 s) were selected from the 12-h continuous recording. Four 5-min segments (Timepoints (TP) 1–4) were analyzed per night and per horse, with two segments (TP1 and TP2) selected from the first half of the night (between 6:00 p.m. and 9:30 p.m.) and two (TP3 and TP4) from the second half (between 1:00 a.m. and 4:15 a.m.). Segments were included only if heart rate was below 60 bpm and no second-degree atrioventricular blocks or movement artifacts were present. This standardized sampling approach ensured a consistent number of high-quality, stationary recordings across horses and nights while providing coverage of the overnight recording period. Manual correction of automated beat detection was performed if needed. The RR intervals of these segments were exported from the Televet software and then imported in the Kubios software (HRV Standard 3.5.0, Copyright ^©^ 2016–2021 Kubios Oy, Finland) for HRV analysis. The following parameters were analyzed: mean heart rate, mean RR intervals, SDNN, RMSSD, SD1, SD2, and LF/HF ratio threshold set at 0.05–0.15 Hz for LF and 0.15–0.5 for HF as defined by Rietmann et al. 2004 (52).

#### 2.7.4 Fecal glucocorticoid metabolite analysis

First, the samples were thawed and weighed and 0.5 g suspended in 4 mL 100% methanol plus 1 mL water, mixed for 30 min and centrifuged (10 min at 2,500 g at room temperature). A second extraction step with diethyl ether was applied as described in detail by Merl et al. (55). For analysis, FCMs were analyzed with an 11-oxoetiocholanolone enzyme immunoassay measuring 11,17-dioxoandrostanes, a group of FCMs (67).

#### 2.7.5 Discomfort score

Item-level scores and total scores were recorded on standardized forms and used for descriptive analysis.

### 2.8 Statistical analysis

Detailed description of the statistical analysis is presented in supplements (Statistical_Analysis_LZ2026.doc). All statistical analyses were performed in R (R version 4.5.2) (68). Significance was declared at 5% cut off. Epochs were characterized regarding sleep stage and lying behavior. Effects on sleep stage probability, sleep cycle probability and other sleep cycle variables (number of sleep cycles, i.e., sleeping frequency, sleep duration per sleep cycle and total sleep duration), lying probability and various lying cycles variables (number of lying cycles, i.e., lying frequency, lying duration per lying cycle and total lying duration), HRV parameters and cortisol metabolites were evaluated via general or generalized linear mixed models. The age class, the interaction between setting and night, and the interaction between setting and time interval were fitted as fixed effects. The horse ID and horse group ID were fitted as random intercepts to account for the covariance structure in the data, as there are multiple measurements per horse and group. Pairwise comparisons between the levels of each categorical predictor were conducted via the estimated marginal means and corrected for multiple testing via the Bonferroni–Holm method.

## 3 Results

### 3.1 Data completeness and technical feasibility

In total 960 h of video recordings were reviewed. Intra-rater variability, evaluated via Domino Software’s Cohen’s kappa, showed an almost perfect agreement.

Due to technical issues, only 868.4 of 960 h of EEG data were successfully annotated; data loss resulted primarily from ganglion disconnections and artifact contamination. Despite minor technical data loss, the EEG mask was generally well tolerated with sufficiently high-quality EEG.

Approximately 868 h of continuous ECG recordings were obtained. For HRV analysis, four artifact-free 5-min segments per horse, night, and housing condition were selected. One week in the shared barn setting for pair D was excluded because of technical difficulties, resulting in a total of 24 h of ECG data included in the final HRV analysis. Two-hundred-fifty-nine of 260 collected fecal samples were analyzed; one sample was lost during transport.

### 3.2 Sleeping behavior

The sleeping behavior was evaluated using all EEG and Video data collected. Across all horses and nights, wakefulness accounted for 77.12% of the recorded time, followed by N2 (9.44%), N1 (5.25%), N3 (4.37%), and REM sleep (3.82%).

**Figure 5:**
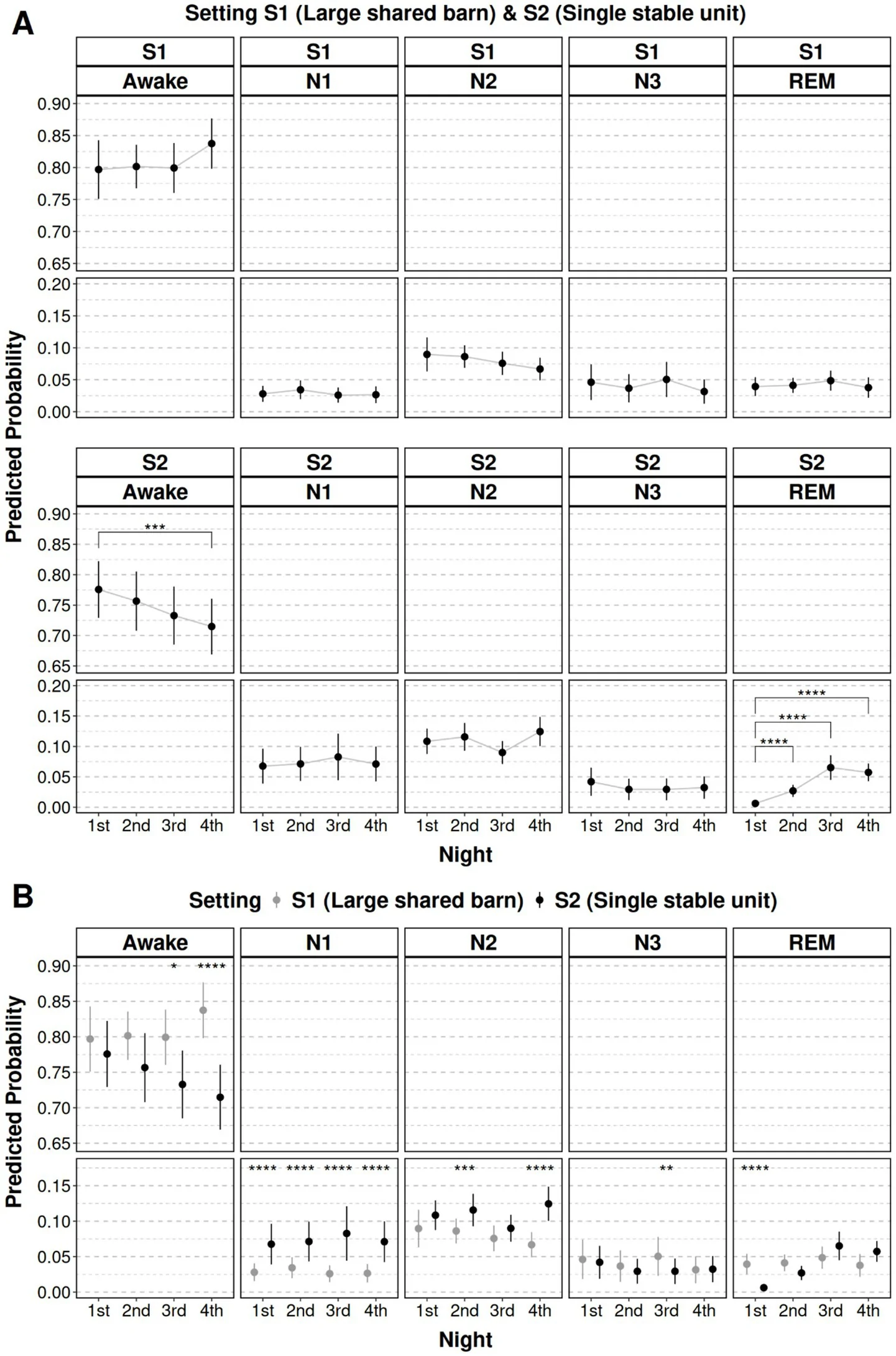
Sleep stage probabilities across settings and nights, evaluated via a multinomial logistic regression mixed model. Estimated marginal means and their 95% confidence intervals were back transformed from the logit scale and are presented as predicted probabilities (response). **(A)** Night differences (for four consecutive nights) for different sleep stages at each setting. Horses in the single stable setting (Setting 2) showed a significant decrease in wakefulness and an increase in REM sleep over time. Results suggest an adaptation to the immediate environment over time. **(B)** Differences between settings (for four consecutive nights) for different sleep stages. Horses in Setting 2 slept more as indicated by wakefulness and light sleep (N1, N2). Multiple testing was accounted for via the Bonferroni–Holm method. Only significant results at a significant cut off of 5% are presented (*p ≤ 0.01, **p ≤ 0.01, ***p ≤ 0.001, ****p ≤ 0.0001).

Comparative analyses revealed clear housing effects. In Setting 2 (individual stalls), horses were significantly less likely to remain awake as nights progressed from the first to the fourth night, (*p* < 0.001), and horses were more likely to exhibit REM sleep over time from the first to the fourth night, (*p* < 0.001) (Figure 5A). Horses housed in the shared barn (Setting 1) demonstrated greater wakefulness and light sleep stages (N1, N2) were more frequently observed (*p* < 0.001) compared to the single stable unit (Setting 2) as the nights progressed (settings contrast at night 4, *p* < 0.001) (Figure 5B).

Throughout the night, more wakefulness was observed within the PM time window (18,00–24,00) and generally more sleep during the AM period (24,00-6,00) (*p* < 0.001 for all sleep stages and settings).

Sleep stages were further grouped into sleep cycles, with a complete sleep cycle consisting of light sleep (N1, N2) and deep sleep stages (N3, REM). This was used to evaluate the occurrence of full sleep cycles, which are cycles consisting of light and deep sleep stages. Further sleep episodes, consisting of only a fraction of sleep stages or even just one sleep stage, were investigated. In Setting 2, when comparing the two time periods (AM and PM), sleep cycles containing light sleep and N3 were more likely to occur during the PM time window (*p* < 0.002), while the probability of cycles consisting only of deep sleep stages was higher during the AM period (however, the latter trend was not significant, *p* = 0.055). The number of sleep cycles was calculated per 100 epochs. During the first night (Day 1), horses in Setting 1 had fewer sleep cycles than those in Setting 2 (*p* = 0.029) - no differences were detected in subsequent nights. In both settings, the number of sleep cycles was higher during the AM period than during the PM period, albeit not significantly (*p* = 0.056). Sleep cycle length varies greatly depending on the composition of the sleep stages. Generally, sleep cycles consisting of multiple sleep stages were longer than those containing only light sleep or deep sleep. Light sleep-only cycles were the shortest, while complete cycles were the longest (*p* < 0.01).

**Figure 6:**
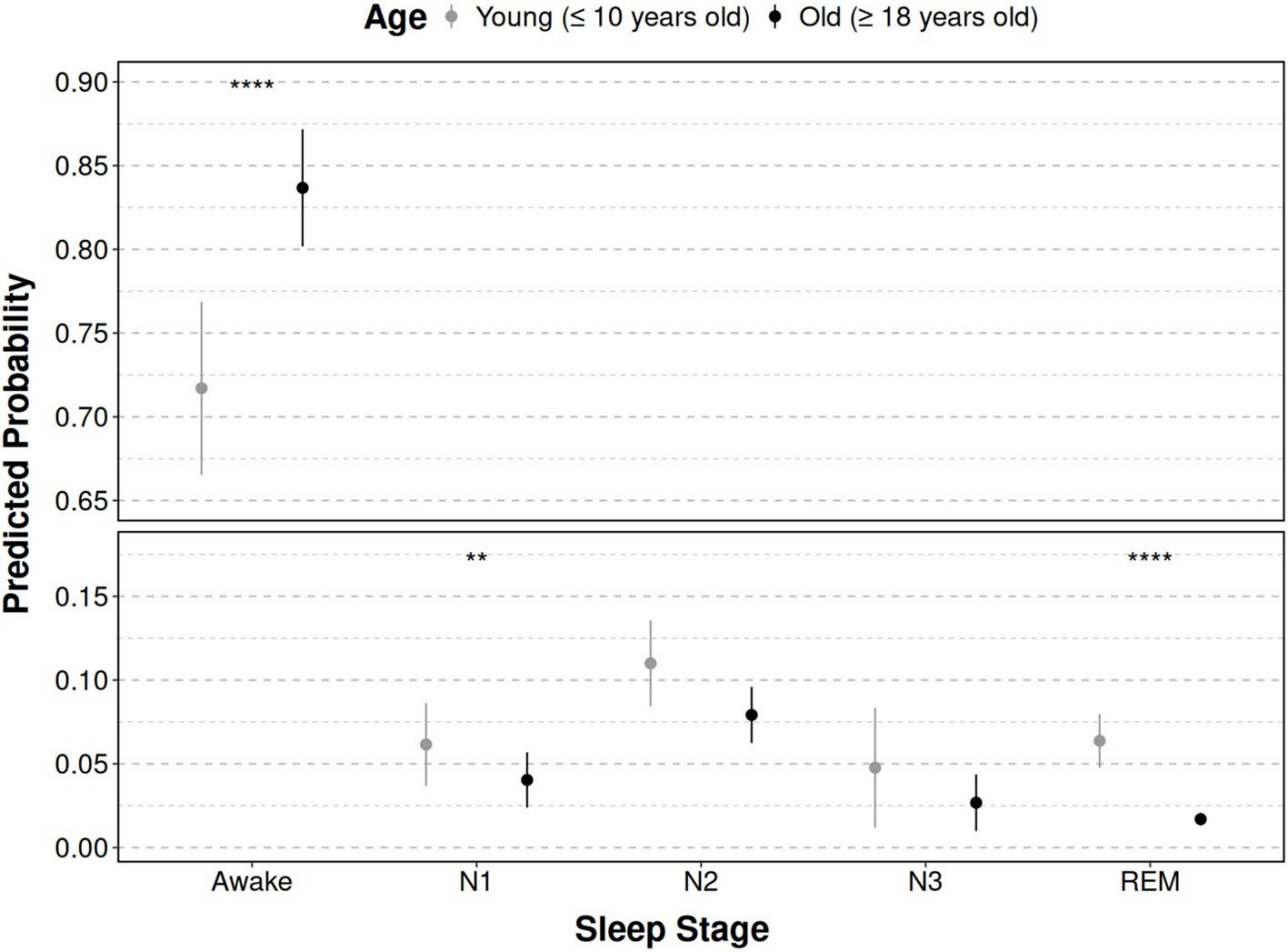
Differences between age classes (young: ≤ 10 years old, old: ≥ 18 years old) for different sleep stages, evaluated via a multinomial logistic regression mixed model. Estimated marginal means and their 95% confidence intervals were back transformed by the logit scale and are presented as predicted probabilities (response). Older horses were more likely to remain awake than younger horses. Multiple testing was accounted for via the Bonferroni–Holm method. Only significant results at a significant cutoff of 5% are presented (*p ≤ 0.01, ****p ≤ 0.0001).

Additionally, age differences were evaluated. Older horses were more likely to remain awake than younger horses (*p* < 0.001). Younger horses exhibited significantly more REM sleep (*p* < 0.001) (Figure 6). Differences were also observed in light sleep stages, with younger horses showing more N1 sleep (*p* = 0.027) and more N2 sleep (*p* = 0.034). The sleep cycles analysis showed that younger horses were more likely to exhibit complete sleep cycles (*p* < 0.001). The analysis of total sleep length in horses also showed an age influence. Younger horses slept longer than older horses (*p* = 0.034).

### 3.3 Lying behavior

The following analysis was based on the complete 960 h of video data; this equals all nights and all horses in both settings. It was considered as lying behavior, when the horses stayed in a lying position rather than lying down immediately rolling and then getting back up. On average, horses laid down 3.55 times for about 2 h and 11 min per night in Setting 1. Similarly, they laid down 3.8 times per night in setting 2, whilst they only spent 1 h and 32 min lying per night in setting 2. To assess lying behavior in detail, analyses of lying probability, lying cycle length, lying cycle rate (number of lying episodes) and total lying duration were performed.

**Figure 7:**
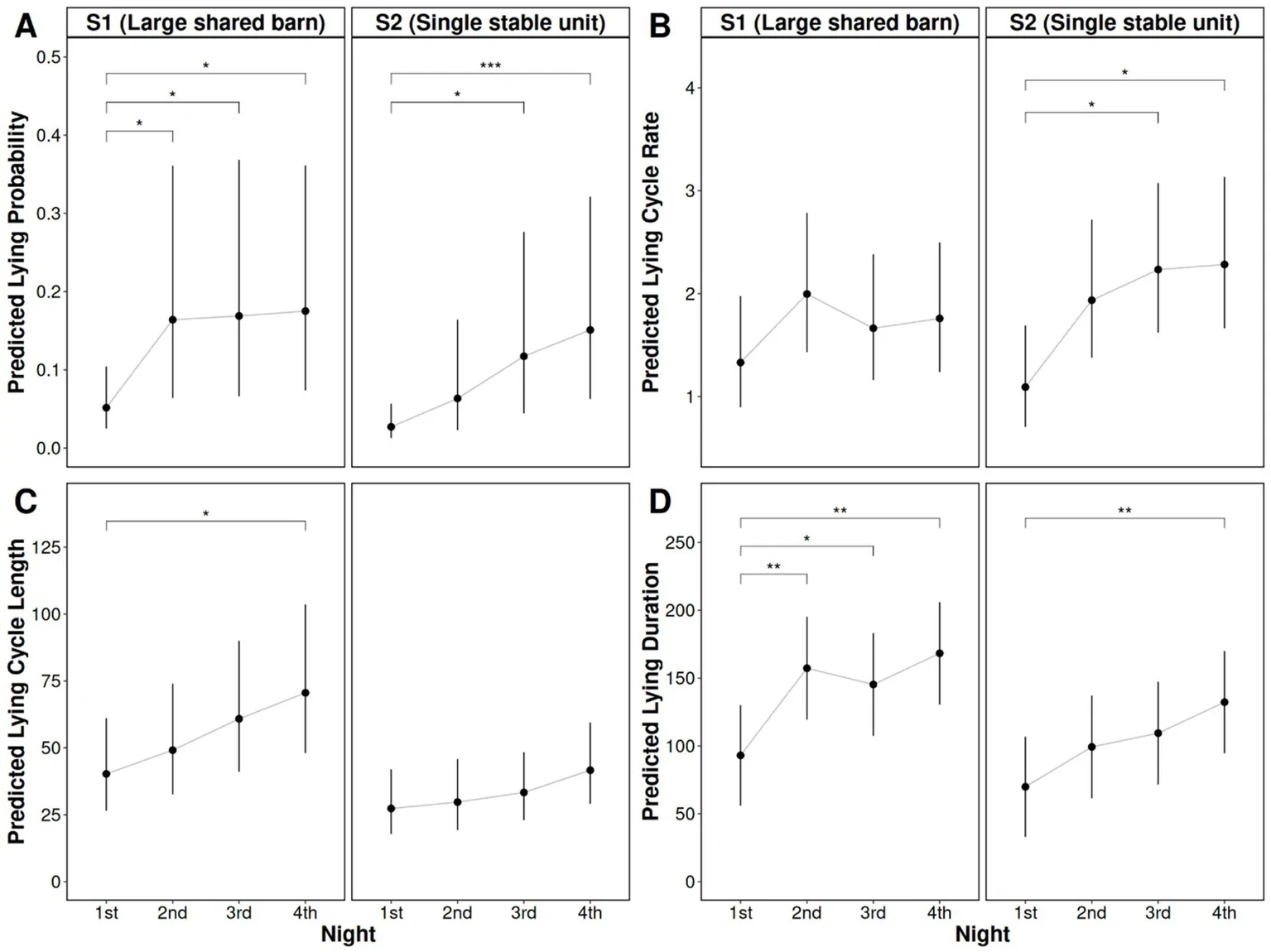
Different aspects of lying behavior across settings and nights. **(A)** Lying probability across settings and nights, evaluated via a binary logistic regression mixed model. Estimated marginal means and their 95% confidence intervals were back transformed from the logit scale and are presented as predicted probabilities (response). **(B)** Number of lying episodes (cycles) across settings and nights, evaluated via a Poisson regression mixed model. Estimated marginal means and their 95% confidence intervals were back transformed from the log scale and are presented as predicted rate (response). **(C)** Duration of lying episodes (lying cycle length) across settings and nights, evaluated via a general linear mixed model. Estimated marginal means and their 95% confidence intervals were back transformed from the log10 scale and are presented on the response scale. **(D)** Estimated marginal means and their 95% confidence intervals for total lying duration across settings and nights. Horses laid down more over time in both settings. Multiple testing was accounted for via the Bonferroni–Holm method. Only significant results at a significant cut off of 5% are presented (*p ≤ 0.01, **p ≤ 0.01, ***p ≤ 0.001).

Significant increasing trends in lying behavior across nights were observed in both settings. The lying probability showed significant increase in both settings from Night 1 to Night 4 (S1: *p* = 0.016, S2: *p* < 0,001) (Figure 7A). There was also a significant increase in the number of lying episodes (cycles) in Setting 2 (Night 1 to Night 4 *p* = 0.027) (Figure 7B). The lying episode (cycle) length across nights gradually increased from Night 1 to Night 4 in Setting 1 (*p* = 0.038) (Figure 7C). A gradual increase of total lying length across nights for both settings from Night 1 to Night 4 was observed (S1: *p* = 0.001, S2: *p* = 0.005) (Figure 7D).

There was also a significant difference in total lying cycle length between the PM and AM periods in Setting 1 (*p* = 0.007), horses lay down significantly longer during the AM period. A similar trend was observed in Setting 2 (*p* = 0.058).

Furthermore, significant differences in the two age groups were identified. Younger horses laid down more frequently compared to older horses (*p* = 0.014), were more likely to have longer lying cycles than older horses (*p* < 0.017) and had greater total lying duration (*p* < 0.005) compared to older horses.

### 3.4 Stress assessment

#### 3.4.1 Heart rate variability analysis

**Figure 8:**
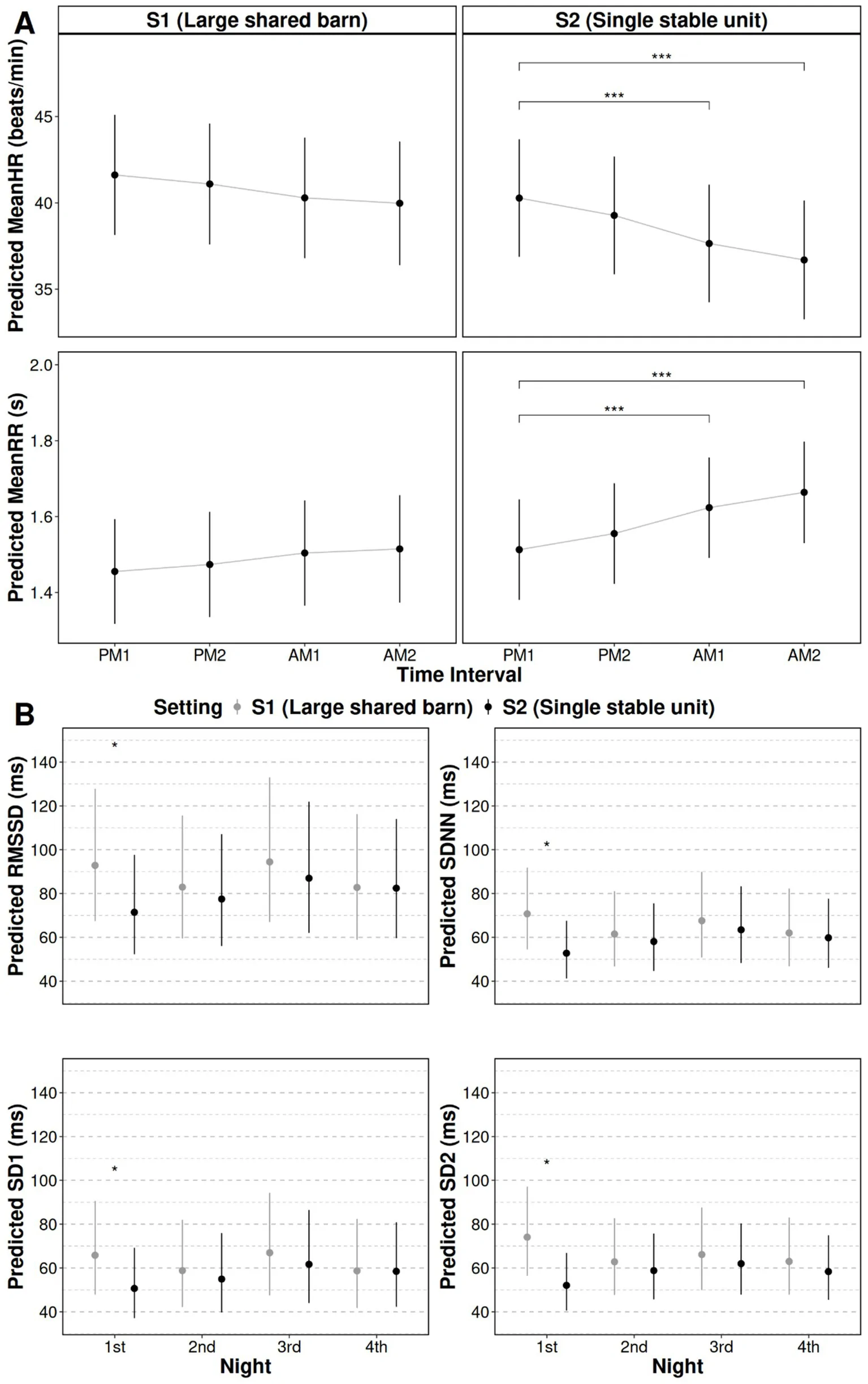
**(A)** Differences between time intervals for mean RR and mean at each setting. Estimated marginal means and their 95% confidence intervals were evaluated via separate general linear mixed models. In Setting 2, mean RR significantly increased from the first to the third and fourth time point, while the mean HR decreased over the same period. **(B)** Differences between settings (for four consecutive nights) for RMSSD, SD1, SD2 and SDNN. Estimated marginal means and their 95% confidence intervals, evaluated via separate general linear mixed models, were back transformed from the log10 scale and are presented as predicted responses. Higher RMSSD, SD1, SD2, and SDNN values indicate more pronounced parasympathetic activity. Multiple testing was accounted for via the Bonferroni–Holm method. Only significant results at a significant cutoff of 5% are presented (*p ≤ 0.01, ***p ≤ 0.001).

In Setting 2, differences in mean RR intervals and mean heart rate were observed in pairwise comparisons between time points (Figure 8A). Between the first and third time points in the night, mean RR intervals significantly increased (*p* < 0,001), and they further increased between the first and fourth time points (*p* < 0,001). The mean heart rate has a strong negative correlation with mean RR intervals (correlation tests not shown); thus the mean heart rate decreased from the first night to the third and fourth night.

The evaluation of setting contrast showed significant differences of HRV variables in the first night. Horses in the shared barn (Setting 1) showed significantly higher RMSSD (*p* = 0.043), SD1 (*p* = 0.043), SD2 (*p* = 0.017) and SDNN (*p* = 0.022) (Figure 8B). No differences were observed in subsequent nights.

#### 3.4.2 Fecal cortisol metabolites

FCM concentrations in between all horses ranged from 1.0 ng/g to a maximum of 20.5 ng/g (Supplementary Table S6). No significant differences were detected in FCM concentrations across nights or settings.

#### 3.4.3 Discomfort scoring

Thirty discomfort scores were obtained (three per horse). Twenty-two scores were 0. Six horses maintained a score of 0 throughout the study. The highest recorded score was 4 and occurred once in a single horse. No horse reached the threshold for moderate or severe discomfort.

## 4 Discussion

### 4.1 Influence of housing environment on sleep and lying behavior

Pronounced differences in sleeping patterns between the two experimental settings were observed. Horses housed in individual stalls (Setting 2) showed a progressive decrease in wakefulness across nights. At the third and fourth night, they were less likely to be awake compared to horses housed in the large, shared barn (Setting1). Conversely, light sleep stages N1 and N2 were observed more often in horses of Setting 2 than those of Setting 1. Furthermore, horses in Setting 2 showed a progressive increase in REM sleep, reaching the same level as in Setting 1. The large, shared barn (Setting1) demonstrated overall greater wakefulness compared to Setting 2. This could be associated with more opportunities for various activities in the large, shared barn setting. The horses were housed in pairs rather than solitary and could interact with other herd members over a fence. Further, there was more space, allowing for more movement as well as more overall possibility for foraging behavior. The differences between settings likely reflect variation in environmental stimulation, bedding characteristics, and social context. Setting 1 provided a large shared lying area with deep litter bedding and opportunities for social interaction and environmental occupation. Previous studies indicate that straw bedding promotes comfort and species-typical foraging behavior (33), and social interaction influences overall activity levels (69–72). This could therefore lead to prolonged lying periods compared to setting 2 as well as increased activity. In contrast, setting 2 offered individual stalls with limited direct social contact and thinner, single-use straw bedding. Reduced bedding depth has been associated with decreased lateral REM sleep and increased standing NREM sleep (8), which may partially explain the shorter recumbency durations observed. However, in this study there was no potentially bedding-associated significant reduction in REM sleep observed in Setting 2 compared to Setting 1.

Differences were also observed in sleep cycle organization and recumbency patterns. Throughout the first night horses showed fewer sleep cycles in the shared large barn (Setting 1) compared to single stable unit (Setting 2), however the sleep cycle length varied depending on its sleep stage composition. Horses in Setting 2 showed overall more light sleep (N1, N2) compared to Setting 1. Deep sleep (N3 and REM) is known to be most restorative, with SWS being linked to physical restoration and REM sleep to cognitive and emotional restoration (5, 10, 73, 74). Therefore, increased sleep duration or sleep cycle length is not necessarily directly linked to increased quality of sleep. Further, while horses in Setting 2 had a significant increase in REM sleep over the 4 consecutive nights, REM sleep occurred relatively consistently in Setting 1. As for the recumbency behavior, probability and total lying duration showed an increase in both settings, however this seems to be achieved in different ways. While in Setting 2 the lying frequency increased overtime, also leading to a significant increase in lying behavior over time, the lying cycle length increases in Setting 1, also leading to a significant increase in lying behavior. This elucidates the different sleeping behavior and adaptation process in the two different settings. This study does not provide additional information into contributing factors for these changes in recumbent behavior. Studies have however previously shown that bedding depth (8), space allowance (75) and herd composition (34) are significant factors when it comes to lying behavior and REM sleep expression. Future studies investigating various lying preferences in association with different management aspects in horses are warranted.

Environmental conditions are known to affect sleep quality in horses. Duncan’s work on Camargue horses demonstrated that disturbances fragmented sleep and reduced REM sleep (76, 77). In the present study, in the single stable setting REM sleep was lowest on the first night and increased thereafter, suggesting that even subtle alterations in routine or environment may disrupt sleep expression.

Since the horses knew Setting 2 from examination or temporary separation from the herd, this could potentially influence initial behavioral responses. Minor observer-related disturbances, such as the presence of recording equipment, could also have contributed to subtle alterations in behavior. Together, these findings emphasize that housing configuration, social context, and bedding conditions collectively shape sleep expression in horses.

Previous studies have reported seasonal differences in resting and recumbent behavior in horses (4), although the contribution of ambient temperature and other seasonal factors remains unclear. Furthermore, these observations have largely been made in horses maintained outdoors (76, 77), whereas access to sheltered resting areas may reduce the influence of environmental conditions on resting behavior (78). The present study was conducted during winter, with ambient temperatures ranging from −6.5 to 12.8 °C, although most observations were recorded between 0 and 10 °C (as seen in Supplementary Table S3). Under these conditions, it is unlikely that ambient temperature substantially influenced the sleep-related behaviors observed, as winter-acclimatized horses are well adapted to maintaining thermal comfort within this temperature range (78, 79). Furthermore, previous research suggests that wind and precipitation represent greater thermoregulatory challenges than ambient temperature alone (78). While high relative humidity has been associated with impaired sleep quality in humans (80), to the authors’ knowledge no studies have investigated its influence on equine sleep. Overall, the direct effects of environmental variables, including ambient temperature, relative humidity, wind and precipitation, on equine sleep remain largely unexplored.

### 4.2 Adaptation across consecutive nights and first-night effects

A clear adaptation effect was observed across nights. HRV analysis showed a significant decrease in Mean HR and increase in Mean RR across nights in Setting 2, this could indicate enhanced parasympathetic activity over time. A similar but non-significant trend was observed in Setting 1. This trend reflects a possible adaptive response overtime (81–83). Further a more pronounced alteration was observed in the sleep and lying behavior, both were reduced on the first night and increased over subsequent nights, particularly in Setting 2. In the single stable unit (Setting 2) REM sleep probability increased significantly, wakefulness decreased, and lying frequency and duration progressively rose from Night 1 to Night 4.

These findings are consistent with the concept of a “first-night effect,” previously described in both humans and animals (15). Dallaire (30) and Oliveira et al. (36) reported that horses modify resting behavior when exposed to environmental changes, often requiring several days to re-establish baseline sleep patterns. Ruckebusch (12) similarly described delayed normalization of recumbency following environmental alterations.

Although both settings were familiar to the horses, minor deviations from their usual routine such as restricted access areas, altered social configuration, or experimental equipment, were sufficient to transiently suppress sleep and recumbency. This highlights the sensitivity of equine sleep behavior to even subtle contextual changes.

The increased sleep expression during the a.m. period aligns with previous reports. Kalus described peak resting phases between midnight and 6 a.m. (24), while Kownacki et al. (84) and Dallaire and Ruckebusch (31) observed comparable nocturnal resting peaks in free-ranging and stabled horses. The present findings support the notion that horses preferentially express consolidated sleep during late-night hours, potentially reflecting reduced environmental disturbance.

### 4.3 Age-related differences in sleep architecture

Age emerged as a significant determinant of sleep expression. Younger horses (5–10 years) exhibited greater total sleep time, higher REM proportions, more complete sleep cycles, and longer recumbency durations compared to older horses (18–26 years). Older horses showed increased wakefulness and had a lower probability for complete sleep cycle, corresponding to more fragmented sleep. These findings are comparable to age related sleep alterations described in humans. In humans, sleep architecture changes substantially across the lifespan, with aging associated with increased fragmentation and reduced REM sleep (18, 85–87). Comparable age-related patterns appear to occur in horses. Although equine sleep research across broad adult age ranges remains limited (25), the present findings suggest that aging may similarly influence sleep consolidation and depth in this species.

The increased sleep expression observed in younger horses may reflect greater restorative or maintenance needs. Sleep has been linked to physical recovery, memory consolidation, and physiological regulation in multiple species (88). While horses reach physical maturity between four and 6 years of age, developmental processes may continue beyond this period depending on breed and management (89). The more fragmented sleep observed in older horses parallels patterns in aging humans, where fragmentation is associated with reduced sleep quality (90).

These findings emphasize the importance of considering age when evaluating equine sleep and welfare.

### 4.4 Physiological stress indicators and their relation to sleep

The significant differences in RMSSD, SD1, SD2, and SDNN between settings suggest differences in autonomic regulation during sleep between the two housing conditions. Higher RMSSD, SD1, and SDNN values in the shared barn (Setting 1) are consistent with greater parasympathetic activity and may reflect lower physiological arousal or greater relaxation. However, these differences were largely confined to the first night, suggesting a transient response during the initial adaptation period. Furthermore, given the potential confounding factors in the study design, including previous experiences associated with the settings, these findings should not be attributed solely to the housing conditions (60, 61, 91).

Increased parasympathetic tone is typically associated with reduced stress and improved physiological regulation, whereas stress is characterized by sympathetic dominance (92, 93). The concurrent increase in REM sleep and recumbency across nights suggests that behavioral adaptation was accompanied by autonomic adjustment. Together, these findings support the hypothesis that acclimation to experimental conditions reduced physiological arousal and promoted sleep expression.

In contrast, FCM concentrations did not differ significantly across nights or settings. This may reflect the delayed nature of FCMs excretion, which integrates hormonal activity over several hours and may be less sensitive to subtle short-term changes (94, 95). Alternatively, environmental modifications induced only mild, transient alterations insufficient to trigger detectable endocrine responses.

Behavioral and physiological assessments indicate low stress levels and absence of clinically relevant discomfort during the study period. Overall, the combined behavioral and autonomic data shows progressive adaptation without evidence of clinically relevant stress.

### 4.5 Implications for husbandry and welfare

Even though horses were familiar with both settings, REM sleep was suppressed on the first night in Setting 2 (single stable units), implying that any change in routine housing may temporarily compromise sleep quality. However, the pronounced first-night effect should be interpreted with caution, as previous experiences associated with Setting 2 and other differences between the experimental settings may also have contributed. The pronounced increase in sleep and lying behavior over four consecutive nights also underscores the potential effect minimal changes in horse’s routine housing have. Any change in the horse’s environment could therefore have similar consequences. This should be considered when relocating horses. Further, the increase in sleep and lying during the early morning hours as previously described (24) underscores once again the importance of minimizing disturbances during this critical time window.

The fact that differences in HRV variables between the two housing settings were largely confined to the first night suggests a housing-related effect during the initial adaptation period, with horses exhibiting indicators of lower stress in the large barn compared to the single-box units. It was reported that individual stabling induces acute and lasting alterations in leukocyte profiles, with welfare-relevant behavioral signs, indicating a negative impact on health and welfare, group housing showed immunocompetence advantages in contrast to single stabling in horses (96). Other studies suggest that individually stabled stallions show impaired social competence and increased aggression when reintroduced to conspecifics (97) and further report that group-housed stallions exhibit higher levels of alertness and social interaction during daily management (98); overall, these findings support group housing as a welfare-enhancing practice.

Young horses sleep more, are more likely to exhibit complete sleep cycles and lie down more frequently and longer than older horses (Quelle). These age-related differences suggest another welfare relevant aspect, for the need for adequate and undisturbed resting opportunities for both age groups. Ensuring appropriate housing design, bedding quality, and social conditions may therefore be particularly important for optimizing welfare.

Moreover, the interplay between sleep, autonomic regulation, and environmental context underscores the value of integrating behavioral and physiological measures when assessing equine welfare.

### 4.6 Limitations

The small sample size (*n* = 10) limits generalizability. Due to technical restraints (Bluetooth transmission range, limited surveillance possibilities) it was not feasible to monitor the horses in their usual enclosure. Setting 1 (large barn setting) was only a part of their open barn enclosure. Therefore, no control setting could be implemented. Settings required different technical setups, with monitors being in the horse’s sight in Setting 2. Thus, the visibility of the first author during routine equipment checks and the presence of monitor lights in Setting 2, but not in Setting 1, may have contributed to differences in the horses’ resting behavior. Additionally, horses were familiar with both experimental settings, which may have attenuated environmental novelty effects compared to completely unfamiliar conditions. Furthermore, although the horses were familiar with both settings, the single-stable units were commonly used for training, teaching activities, and periods of isolation. As a result, the horses may have associated these environments differently, indicating that the quality and meaning of their experiences were not equivalent across the two surroundings. Nevertheless, the presence of first-night alterations demonstrates that even subtle deviations within familiar environments can influence sleep expression.

A potential limitation of the present study is the influence of environmental conditions on sleep behavior and physiological parameters. To minimize such effects, all measurements were conducted within the same season, thereby reducing variability associated with substantial changes in weather conditions. Furthermore, each horse pair underwent experimental conditions within consecutive weeks, separated by a washout period, to limit temporal variation between setting exposures. Ambient temperature was recorded throughout the study and remained comparable between measurement periods (as shown in Supplementary Table S3). However, relative humidity was not monitored and therefore could not be considered in the analysis. As humidity may influence thermal comfort and resting behavior, its potential contribution to the observed outcomes cannot be entirely excluded. Nevertheless, the standardized study schedule and restricted study period were intended to minimize environmental variation and improve comparability between measurements.

Even though HRV and cortisol-related measures provide objective insights into stress response, they lack real-time applicability and may not fully capture acute stress-related alterations in sleep. Equine EEG methodology remains a developing field, and standardized scoring criteria are not yet fully established. Although data quality was sufficient for analysis, technical losses occurred. Further validation studies and larger sample sizes are warranted to refine methodological approaches and establish normative reference values.

## 5 Conclusion

In conclusion, horses exhibited increased REM sleep, lying probability, and total lying time across nights, suggesting an adaptation effect. Our results on HRV suggest trends toward increased parasympathetic activity, which supports a possible acclimation. Further the difference in HRV variables could indicate a stress response in the single stable setting. Sleep was more prevalent during a.m. period than during the p.m. period. Younger horses experienced more REM sleep, more complete sleep cycles and longer lying durations compared to older horses. These results underscore the significance of environmental and age-associated influences on equine sleep and welfare, reinforcing the need for management approaches that promote adequate restorative rest. Further investigation is warranted to validate EEG methodologies in equine medicine, including the characterization of electroencephalographic features in horses sleep medicine and the integration of electrocardiographic parameters, as well as to elucidate the relationship between sleep and overall welfare.

## Data Availability

The original contributions presented in the study are included in the article/Supplementary material, further inquiries can be directed to the corresponding author.
