# Correction: Sleep architecture and lying behavior in horses across age groups and two housing systems: an overnight non-invasive EEG study

**DOI:** 10.3389/fvets.2026.1981722

**Published:** 2026-09-16

**Authors:** Frontiers Production Office

**Affiliations:** Frontiers Media SA, Lausanne, Switzerland

**Keywords:** equine age impact, equine EEG, equine sleep, equine welfare, first-night-effect

The funder University of Veterinary Medicine Vienna was erroneously omitted from the Funding statement. The correct **Funding** statement reads:

“The author(s) declared that financial support was received for this work and/or its publication. This work was co-founded by the Austrian Research Funding Agency (FFG), the Austrian Federal Ministry of Science, Research and Economy (BMWFW). Open access funding provided by University of Veterinary Medicine Vienna.”

The original version of this article has been updated.

