# Supplementary material for: Sleep architecture and lying behavior in horses across age groups and two housing systems: an overnight non-invasive EEG study
Source: Front Vet Sci. 2026 Aug 6;13:1898580. doi: 10.3389/fvets.2026.1898580 (PMC13491436; doi:10.3389/fvets.2026.1898580)
Supplement: Supplementary file 1 [file Data_Sheet_1.pdf]

Statistical analysis

All statistical analyses were performed in R (R version 4.5.2) (R Core Team 2025). Significance was declared at 5% cut off. ggplot2 was used for visualization of the results (package *ggplot2*, Version 3.5.2, function *ggplot*) (Wickham 2016). Epochs were characterized in regard to sleep stage and lying behavior. 15 Rem epochs for which the horse was standing changed to awake (for 7 cases, the horse was awake and standing for several epochs after the changed epoch, while the 8 remaining cases correspond to successive epochs of a specific horse and fall between several awake and standing epochs). Below we list all evaluated response variables and the models that were implemented.

<u>Sleep stage probability</u>

Effects on the probability of each sleep stage were evaluated via a multinomial logistic regression mixed model (package *mclogit*, version 0.9.6, function *mblogit*, options 'control = mmclogit.control(maxit = 10000, trace = T)') (Elff 2022). The sleep stage was fitted as a categorical response with 5 levels (Awake, N1, N2, N3, Rem).

<u>Sleep cycle probability</u>

Sleep cycles were also recorded. If there were at most two 'awake' epochs and/or epochs missing, the sleep cycle did not end. Based on the included sleep stages, sleep cycles were characterized as:

a) Only light sleep: Cycle contains stages N1 and/or N2 only
b) Light sleep with N3: Cycle contains stages N1 and/or N2 together with stage N3
c) Light sleep with REM: Cycle contains stages N1 and/or N2 together with stage REM
d) Only deep sleep: Cycle contains stage N3 only or stage REM only
e) Complete: Cycle contains stages N3 and REM together with stages N1 and/or N2

Effects on the probability of each sleep cycle were evaluated via a multinomial logistic regression mixed model as described above. This model did not include cycles with duration of 1 epoch.

<u>Other sleep cycle variables</u>

Effects on the frequency of sleeping (number of sleeping cycles), sleep duration per sleep cycle (in epochs) and total sleep duration (in epochs) were evaluated via separate general linear mixed models (package *lme4*, function *lmer*, Version 1.1.37, options 'REML = FALSE, control = lmerControl(optimizer = "bobyqa", optCtrl = list(maxfun = 10000))') (Bates et al. 2015). Number

of sleeping cycles and total sleep duration were expressed per 100 epochs to account for missing data that result in different number of observation per horse, setting, night and time interval. Sleep duration per sleep cycle was log10-transformed. These models did not include cycles with duration of 1 epoch.

Lying probability

Effects on the probability of lying were evaluated via a binary logistic regression mixed model (package *lme4*, function *glmer*, option 'family = binomial (link = "logit"), control = glmerControl(optimizer = "bobyqa", optCtrl = list(maxfun = 10000)), nAGQ = 0').

Frequency of lying

Lying cycles were also recorded (as described above for sleep cycles). Effects on the frequency of lying (number of lying cycles) was evaluated via a poisson regression mixed model (package *lme4*, function *glmer*, option 'family = poisson(link = "log"), control = glmerControl(optimizer = "bobyqa", optCtrl = list(maxfun = 10000))').

Other lying cycle variables

Effects on lying duration per lying cycle (in epochs) and total lying duration (in epochs) were evaluated via separate general linear mixed models as described above. Lying duration per lying cycle was log10-transformed.

HRV parameters

Effects on heart rate variability parameters:

- High Frequency activity [HF(Hz), HF (n.u.)],
- Low frequency activity [LF(Hz), LF (n.u.)],
- Mean Heartrate [MeanHR(beats/min)],
- Mean RR interval [MeanRR(ms)],
- Root Mean Square of Successive Differences [RMSSD (ms)],
- Standard Deviation of NN intervals [SDNN (ms)]

- Short-term variability parameter SD1 for short term quantification of beat to beat variability and
- Long-term variability parameter (SD2) the standard deviation of the Poincare plot along the line of identity
- SD1/SD2 ratio

were evaluated via separate general linear mixed models as described above. Mean RR-interval was divided by 1000 (to be expresses in seconds) and RMSSD, SD1, SD2 and SDNN were log10-transformed.

<u>Cortisol metabolites</u>

Effects on cortisol metabolites were evaluated via a general linear mixed model as described above. Cortisol metabolite values were log10-transformed. Average morning and evening baseline (log10-transformed) cortisol metabolite values were included in the model as a covariate.

<u>Structure of these models</u>

The age class (2 age classes, horses were classified as younger than 11 years or older than 17 years of age), the interaction between setting (2 settings) and night (4 nights) and the interaction between setting (2 settings) and time interval (2-time intervals, 18:00 – 24:00 and 00:00 – 06:00) were fitted as fixed effects. The model for sleep duration per sleep cycle further included the cycle characterization as a fixed effect. For HRV parameters, there were four-time intervals (2 3-hour intervals before and 2 3-hour intervals after midnight). The horse ID (10 horses) and horse group ID (5 groups, groups C and F were combined) were fitted as random interceptions to account for the covariance structure in the data, as there are multiple measurements per horse and group. Furthermore, since each horse and group were examined multiple times at each setting, night and time interval, the interactions between dummy coded centered setting and nights and the interaction between dummy coded centered setting and night interval were fitted as random slopes within horse ID and horse group ID.

<u>Further analysis</u>

Assumptions about the residuals, random intercepts and random slopes were visually evaluated. Multicollinearity between the fixed effects was evaluated via variance-inflation factors (package

car, version 3.1.3, function *vif*) (Fox and Weisberg 2019) on models without interactions. Stability of each model was evaluated with a leave-one-out strategy by comparing fitted values based on the model using all data to those of models based on data with horse IDs or horse group IDs (random effects) excluded one at a time. For the poisson regression mixed model, overdispersion was also evaluated (package *performance*, version 0.13.0, function *check_overdispersion*) (Lüdecke et al. 2021).

Pairwise comparisons between the levels of each categorical predictor were conducted via the estimated marginal means (package *emmeans*, version 1.11.0, functions *emmeans*, *regrid* and *pairs*) (Lenth 2025). For presentation, estimated marginal means were back transformed (when applicable, e.g. from the *log10* or *logit* scale, option 'type = "response"' in the function *emmeans*). Multiple pairwise comparisons of nights to the first night only at each setting, between setting at each night and between time intervals at each setting were separately corrected for multiple testing via the Bonferroni–Holm method (function *p.adjust*).

References

Bates, Douglas, Martin Mächler, Benjamin M. Bolker, and Steven C. Walker. 2015. "Fitting Linear Mixed-Effects Models Using Lme4." *Journal of Statistical Software* 67 (1): 1–48. https://doi.org/10.18637/jss.v067.i01.

Elff, Martin. 2022. "Mclogit: Multinomial Logit Models, with or without Random Effects or Overdispersion." *R Package Version 0.9.6*. https://cran.r-project.org/package=mclogit.

Fox, John, and Sanford Weisberg. 2019. "An {R} Companion to Applied Regression, Third Edition." *Thousand Oaks CA: Sage.* https://socialsciences.mcmaster.ca/jfox/Books/Companion/.

Lenth, Russel V. 2025. "Emmeans: Estimated Marginal Means, Aka Least-Squares Means." *R Package Version 1.11.0*. https://cran.r-project.org/package=emmeans.

Lüdecke, Daniel, Mattan Ben Shachar, Indrajeet Patil, Philip Waggoner, and Dominique Makowski. 2021. "Performance: An R Package for Assessment, Comparison and Testing of Statistical Models." *The Journal of Open Source Software* 6 (April): 3139. https://doi.org/10.21105/joss.03139.

R Core Team. 2025. "R: A Language and Environment for Statistical Computing." *R Foundation for Statistical Computing, Vienna, Austria*. https://www.r-project.org/.

Wickham, Hadley. 2016. "Ggplot2: Elegant Graphics for Data Analysis." *Springer-Verlag New York*. https://ggplot2.tidyverse.org/.
