# Supplementary material for: Sleep architecture and lying behavior in horses across age groups and two housing systems: an overnight non-invasive EEG study
Source: Front Vet Sci. 2026 Aug 6;13:1898580. doi: 10.3389/fvets.2026.1898580 (PMC13491436; doi:10.3389/fvets.2026.1898580)
Supplement: Supplementary file 2 [file Data_Sheet_2.pdf]

Supplementary Material

Supplementary Figures and Tables

Table S1: Horses included in the study, grouped according to pairs in alphabetical order (A1 and A2, B1 and B2 etc.)

Horse	Sex	Age	Breed
A1	mare	20	Haflinger
A2	mare	24	Haflinger
B1	mare	26	Haflinger
B2	mare	21	Haflinger
C2	mare	18	Trotter
F1	gelding	10	Trotter
D1	mare	5	Haflinger
D2	mare	5	Haflinger
E1	gelding	22	Trotter
E2	mare	6	Haflinger

Table S2: Impedance measurements from all horses (A1-E2) in all nights and settings at start and finish of the recording time.

Horses	S1D1		S1D2		S1D3		S1D4		S2D1		S2D2		S2D3		S2D4	
	Start	End	Start	End	Start	End	Start	End	Start	End	Start	End	Start	End	Start	End
**A1**																
Ch 1	2	17	2		0.5	0.5	2	1	6	5	6.5	10.5	5.5	25.5	0.5	1.5
Ch 2	1.5	38.5	2.5		3	0.5	6	2	8.5	6	6	9	4	17	9	17.5
Ch 3	3	15.5	1		3.5	0.5	2	5	2.5	1.5	6.5	7.5	3	15	5	4.5
Ch 4	1.5	16	2		2.5	0.5	3	479.5	3	1	6.5	12	3	7.5	4.5	4
Ref	4.5	22	5.5		1.5	0.5	7.5	5	3.5	2.5	6.5	6	5	5	5.5	5
**A2**																
Ch 1	18	17.5	1		0.5		1.5		3.5	2	0.5	0.5	0.5	0.5	1	0.5
Ch 2	40	40	1.5		0.5		0.5		0.5	2	0.5	0.5	0.5	0.5	1	0.5
Ch 3	17	16.5	1		1		386.5		0.5	1.5	0.5	0.5	0.5	0.5	1	2
Ch 4	17.5	17	0.5		0.5		0.5		0.5	1.5	2.5	4.5	2	0.5	5.5	3.5
Ref	23	23	1.5		0.5		0.5		0.5	2	0.5	0.5	0.5	0.5	0.5	0.5
**B1**																
Ch 1	3	25.5	6	4	5	3	2.5	2	26.5	15.5	5.5	11	4	11.5	3	9
Ch 2	1.5	4	7.5	2.5	6	2	1.5	1.5	32	138	2	38.5	4	10.5	4	11
Ch 3	4.5	14	8.5	3	3	1.5	2	1	15	6.5	0.5	603.5	3	11.5	2	9.5
Ch 4	24.5	12.5	5	13	3	2	8	2	25	16	2	7.5	3	12	2.5	7.5
Ref	2.5	5.5	18.5	2	5	2.5	2	1.5	25	9	1	8	4	14	4.5	18.5
**B2**																
Ch 1	4	12	1.5	2.5	5	3	3	3.5	5	386.5	7.5	13	4	11.5	2.5	9
Ch 2	5	14.5	2.5	1.5	6	2	3	4.5	5	479.5	7.5	12	4	10.5	2.5	11
Ch 3	3	11.5	1.5	1	3	1.5	3	3.5	5.5	479.5	8	12	3	11.5	2.5	9.5
Ch 4	3.5	12.5	1.5	2	3	2	2.5	3.5	5.5	479.5	7	12	3	12	2	7.5
Ref	4.5	13	2	1	1	2.5	3	2.5	6.5	479.5	8	14	4	14	4.5	18.5
**C2**																
Ch 1	1	6	0.5	4	0.5	0.5	0.5	0.5	4	1	0.5	0.5	0.5	0.5	1.5.	1
Ch 2	1	2	0.5	2	0.5	0.5	1	0.5	2.5	1	0.5	0.5	1.5	0.5	1.5	0.5
Ch 3	0.5	2	0.5	3	0.5	0.5	0.5	0.5	0.5	0.5	0.5	0.5	0.5	0.5	1.5	1.5
Ch 4	0.5	2	0.5	3	0.5	1	0.5	0.5	0.5	0.5	0.5	0.5	0.5	0.5	0.5	0.5
Ref	1	1.5	1	3.5	0.5	2.5	0.5	1.5	1	1.5	0.5	0.5	0.5	1	0.5	1.5
**F1**																
Ch 1	1.5	1.5	0.5	1.5	0.5	10	0.5	13.5	0.5	12.5	0.5	1	0.5	0.5	0.5	1.5
Ch 2	1.5	2	0.5	1.5	0.5	7	0.5	12	0.5	12.5	0.5	1	0.5	0.5	0.5	1
Ch 3	1.5	1.5	0.5	1.5	0.5	7.5	0.5	10.5	0.5	13	0.5	1	0.5	0.5	0.5	1
Ch 4	1.5	1.5	0.5	2	0.5	10.5	0.5	10.5	1	12	0.5	1	0.5	0.5	0.5	1.5
Ref	1	2	0.5	4	0.5	8	1	9.5	0.5	32	0.5	2	0.5	0.5	0.5	1.5
**D1**																
Ch 1	0.5	121	0.5	0.5	1.5	5.5	1	2.5	2.5	5	0.5	1	0.5	2.5	0.5	0.5
Ch 2	1.5	18.5	0.5	3	2	4	1	2	1.5	159	0.5	0.5	0.5	38.5	0.5	0.5
Ch 3	1.5	14.5	0.5	1	2.5	6.5	1.5	2	2	6	0.5	0.5	0.5	1	0.5	0.5
Ch 4	1	17.5	0.5	1	1	7.5	1	2	2	6	0.5	1	0.5	43.5	0.5	0.5
Ref	1	16.5	0.5	0.5	3.5	5.5	1.5	3.5	2	5.5	0.5	0.5	0.5	1	0.5	0.5
**D2**																
Ch 1	1	1.5	0.5	0.5	0.5	1.5	1	1	1	603.5	0.5	0.5	0.5	4	0.5	603.5
Ch 2	1.5	1.5	1	1	1	2	0.5	1	0.5	479.5	1	0.5	0.5	1.5	0.5	603.5
Ch 3	0.5	1	0.5	0.5	0.5	0.5	0.5	0.5	1	603.5	0.5	0.5	0.5	1.5	0.5	603.5
Ch 4	1.5	1	0.5	0.5	1	46	1	0.5	1.5	479.5	0.5	0.5	0.5	3	0.5	603.5
Ref	1.5	1.5	0.5	0.5	0.5	0.5	0.5	0.5	1.5	603.5	2	0.5	3	6	1	479.5
**E1**																
Ch 1	1	6	0.5	4	0.5	0.5	0.5	0.5	4	1	0.5	0.5	0.5	0.5	1.5	1
Ch 2	1	2	0.5	2	0.5	0.5	1	0.5	2.5	1	0.5	0.5	1.5	0.5	1.5	0.5
Ch 3	0.5	2	0.5	3	0.5	0.5	0.5	0.5	0.5	0.5	0.5	0.5	0.5	0.5	1.5	1.5
Ch 4	0.5	2	0.5	3	0.5	1	0.5	0.5	0.5	0.5	0.5	0.5	0.5	0.5	0.5	0.5
Ref	1	1.5	1	3.5	0.5	2.5	0.5	1.5	1	0.5	0.5	0.5	0.5	1	0.5	1.5
**E2**																
Ch 1	1.5	1.5	0.5	1.5	0.5	10	0.5	13.5	0.5	12.5	0.5	1	0.5	0.5	0.5	1.5
Ch 2	1.5	2	0.5	1.5	0.5	7	0.5	12	0.5	12.5	0.5	1	0.5	0.5	0.5	1
Ch 3	1.5	1.5	0.5	1.5	0.5	7.5	0.5	10.5	0.5	13	0.5	1	0.5	0.5	0.5	1
Ch 4	1.5	1.5	0.5	2	0.5	10.5	0.5	10.5	1	12	0.5	1	0.5	0.5	0.5	1.5
Ref	1	2	0.5	4	0.5	8	1	9.5	0.5	32	0.5	2	0.5	0.5	0.5	1.5

Table S3: Night allocation schedule: Data collection took place from 26.12.22 through to 3.3.23. The first night of setting exposure and electrophysiological data recording for each pair always being from Monday to Tuesday and the fourth therefore from Thursday to Friday. Recording periods of 12 hours were set from 6pm to 6am. Setting in which pair commenced was randomly assigned.

Time of data collection	Pair	Setting
Week 1	A	S1
Week 2	A	S2
Week 3	B	S2
Week 4	B	S1
Week 5	C/F	S1
Week 6	C/F	S2
Week 7	D	S2
Week 8	D	S1
Week 9	E	S1
Week 10	E	S2

Table S4: Included epochs per horse/night/setting. One epoch equals 30seconds.

	Included Epochs per Horse and Condition									
Settings &	Pair A		Pair B		Pair C/F		Pair D		Pair E	
Nights	A1	A2	B1	B2	C	F	D1	D2	E1	E2
S1D1	1088	1138	1429	1154	931	1289	1432	1274	1113	1424
S1D2	132	1369	1424	1437	1444	1212	1264	1396	1225	1425
S1D3	0	1025	1400	1399	1082	1393	1421	1417	1286	1343
S1D4	0	1418	1415	1407	1445	1119	1429	1413	1440	1434
S2D1	1429	1438	1388	1272	1404	1398	1418	1020	1416	1413
S2D2	1409	1420	1433	1426	1396	1404	1384	1451	1450	1460
S2D3	1436	1425	1423	1421	1415	1425	1210	1439	1431	1443
S2D4	1291	1448	1420	1416	1385	1397	1436	823	1429	1383

Table S5: Faecal Cortisol Metabolites from all horses (A1-E2), sampled during setting exposure (S1, S2) and at baseline time (B), at am and pm timepoints in nights (N0-N4).

			Cortisol Metabolites ng/g									
Setting	Night	Time	A1	A2	B1	B2	C1	F2	D1	D2	E1	E2
S1	N0	am	4.3901	5.7574	10.5512	4.4264	4.5705	8.2478	3.6938	3.1999	4.1206	4.9258
S1	N0	pm	2.5454	3.1889	8.9023	4.3461	2.8897	3.5728	2.6158	2.7555	2.6411	4.0634
S1	N1	am	2.629	5.7552	11.8151	3.9974	2.2858	2.6521	2.9777	3.0008	3.1801	5.7475
S1	N1	pm	2.7544	8.8264	13.0603	6.0445	2.9546	5.698	5.3163	4.8279	4.037	10.604
S1	N2	am	2.4266	6.5703	7.9046	6.6341	2.893	4.9907	4.8477	3.2461	2.1285	6.0786
S1	N2	pm	3.1944	4.7355	6.2843	4.6189	1.9866	6.776	3.9688	3.3055	2.3485	5.2679
S1	N3	am	3.2318	6.0841	9.2367	4.7278	2.3001	8.3963	2.519	2.8534	5.1007	4.0062
S1	N3	pm	3.2263	6.9465	4.8323	5.0446	2.6323	5.907	2.5597	3.0371	1.6005	6.9509
S1	N4	am	3.4463	4.8488	12.4168	7.0466	2.1406	5.3999	1.4245	3.4628	3.7026	4.0568
S1	N4	pm	3.0943	4.466	8.3072	6.8409	3.1592	7.865	3.7532	5.2756	2.9304	4.6915
S2	N0	am	2.4651	6.4438	15.1965	5.8564	4.2471		2.4068	2.9139	2.1131	4.0876
S2	N0	pm	3.3517	4.9445	9.8384	5.8927	3.9732	6.6924	3.9919	5.4373	2.5949	4.7729
S2	N1	am	3.9402	3.7774	9.0288	5.1359	3.7488	7.6791	3.0712	8.1719	4.2218	5.3636
S2	N1	pm	3.0316	4.1305	15.8664	6.2469	3.1526	5.8993	2.7687	5.995	2.6026	4.2779
S2	N2	am	4.3472	10.373	20.5656	6.4361	3.08	8.0784	3.2483	3.9974	1.5169	3.2076
S2	N2	pm	4.246	1.8568	17.2282	5.6155	4.7058	9.0178	3.9633	5.1898	2.3034	5.4791
S2	N3	am	4.7817	6.9531	10.1277	4.9082	3.2736	5.0303	2.8644	4.4363	1.5169	4.4
S2	N3	pm	3.6839	2.1076	5.434	5.8443	4.2471	7.8177	4.3901	4.3516	1.5169	5.2008
S2	N4	am	3.1383	3.6971	7.7539	5.511	5.4494	6.7089	4.5199	6.5549	2.5696	4.3406
S2	N4	pm	2.3243	3.4793	5.6001	5.5506	2.8765	5.4131	3.2164	4.8301	2.1274	4.2394
B	B1	am	1.9536	1.5862	11.1276	5.8597	2.409	9.3456	3.0063	4.4297	2.5641	3.256
B	B1	pm	2.1879	4.7014	12.0648	5.3691	3.1163	7.0422	2.5751	6.347	3.6597	4.6431
B	B2	am	1.5763	7.9728	11.5324	6.4042	2.1505	7.1522	6.9817	3.6498	2.9568	3.8445
B	B2	pm	2.4981	4.8235	10.7404	8.0564	1.6654	6.7694	2.6257	4.6794	2.7423	5.0523
B	B3	am	2.497	2.6213	12.9569	5.7596	2.1934	2.893	4.2185	4.6794	3.9677	6.7177
B	B3	pm	1.0241	5.1964	17.6946	11.6169	4.6376	3.6289	3.1042	5.3812	2.2484	6.5494

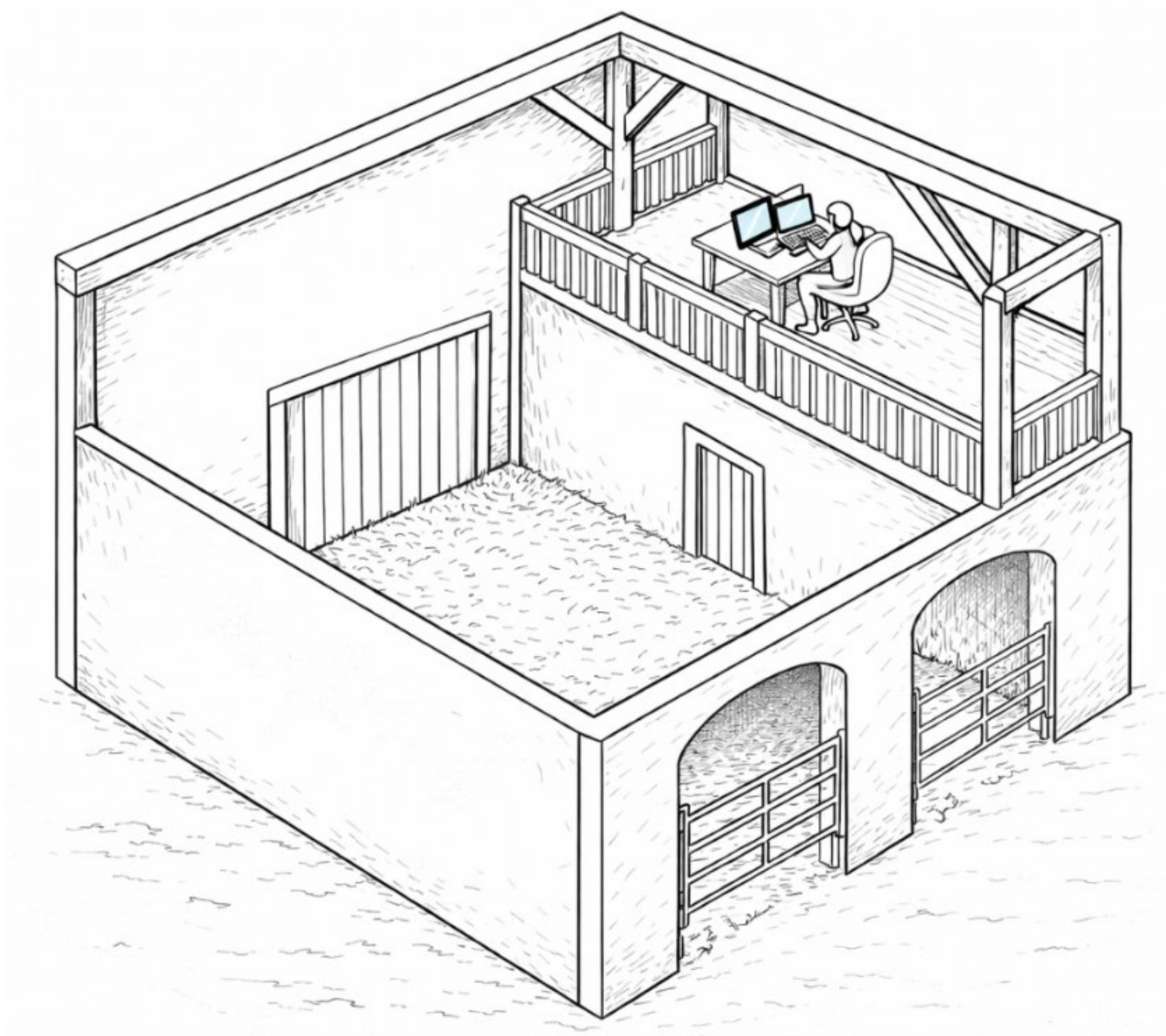

Figure S1: Schematic drawing generated by Chatgpt from setup at Setting 1.

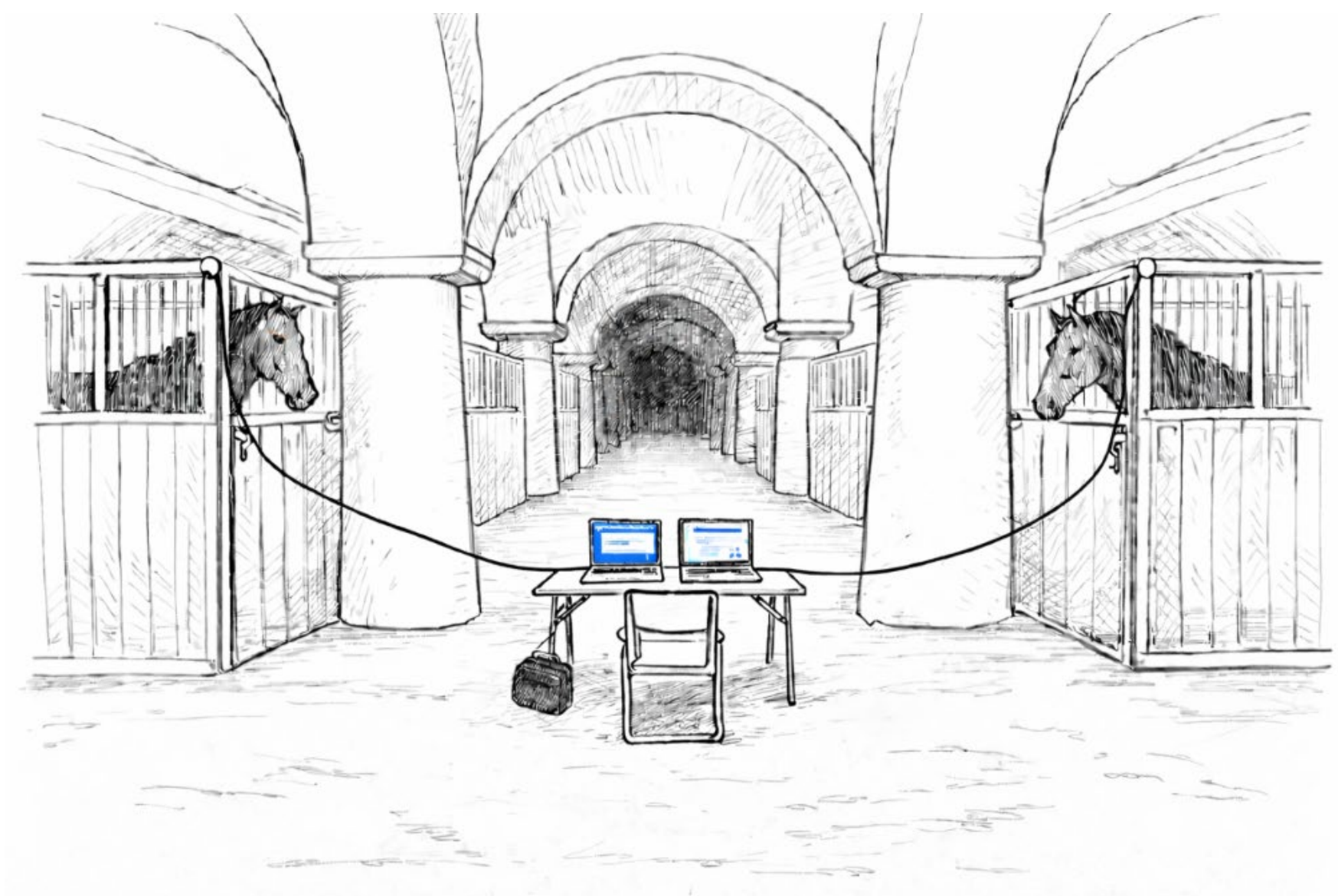

Figure S2: Schematic drawing generated by Chatgpt from setup at Setting 2.
